# Effect of Grafting Density on the Crystallization Behavior of Molecular Bottlebrushes

**DOI:** 10.1021/acs.macromol.4c00752

**Published:** 2024-08-20

**Authors:** Jeffrey T. Wilk, Carl T. Furner, Ethan W. Kent, Michael T. Kelly, Bin Zhao, Christopher Y. Li

**Affiliations:** † Department of Materials Science and Engineering, Drexel University, Philadelphia, Pennsylvania 19104, United States; ‡ Department of Chemistry, University of Tennessee, Knoxville, Tennessee 37996, United States

## Abstract

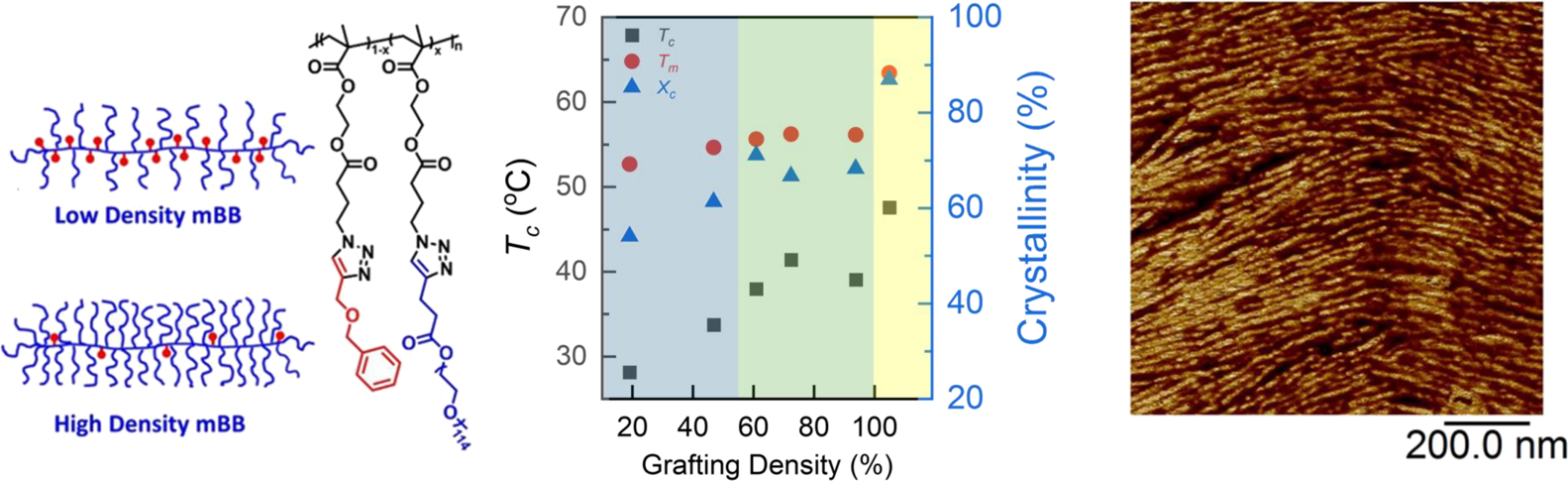

A unique case of sterically constrained crystallization arises in bottlebrush polymers bearing semicrystalline side chains. Bottlebrushes with grafted side chains can form crystalline structures governed by the complex interplay between side chain packing and backbone confinement. The confinement effect can be readily tuned by varying the side chain grafting density, thus affording control over the crystallization behavior of these systems. In this work, the grafting density effect on the crystallization behavior of molecular bottlebrushes comprising poly(ethylene oxide) (PEO) side chains grafted to a methacrylate backbone was systematically studied. Thermal analysis using differential scanning calorimetry showed that the bottlebrush polymers displayed suppressed crystallization temperatures, lower melting temperatures, and reduced crystallinities compared to linear homopolymer PEO. The crystalline morphology was investigated using polarized light, atomic force, and scanning electron microscopy. Isothermal crystallization experiments revealed a nonmonotonous dependence of the nucleation density on the side chain grafting density. The grafting density effect was also investigated using self-seeding experiments, revealing an increased clearing temperature and memory retention at higher grafting densities. This work highlights how grafting density influences the crystallization behavior of semicrystalline bottlebrushes, providing information for the processing and application of these unique polymers.

## Introduction

Molecular bottlebrushes (mBBs) represent a unique type of polymers containing side chains grafted to a backbone.^1−4^ The neighboring side chains strongly repel one another, orienting them normal to the backbone. The increased steric hindrance of the side chains, in turn, forces the backbone to extend, increasing its persistence length.^4−6^ The architecture of the molecular bottlebrushes has a significant influence on the properties of the system. Extensive research has been performed on mBB materials owing to their unique properties, with some notable applications being lubrication,^7−9^ emulsification,^10,11^ electronics,^12^ and drug delivery.^13^ mBB properties can further be influenced through introduction of semicrystalline side chains onto the brush backbone. The crystallization behavior of mBBs bearing crystalline side chains often differs from linear polymers due to the tethering and confinement effect. Yu-Su et al. reported a suppression of crystallization and a shift toward lower crystallization temperature (*T*_c_) in bottlebrushes with block copolymer side chains.^14^ Shish-kebab-like structures with distinct crystalline and amorphous regions were observed.^14^ Sun and co-workers reported how the backbone and spacer moieties can act as defects toward crystallization, increasing nucleation at the cost of radial growth rate.^15^ Wu et al. synthesized a series of poly(ethylene glycol) with increased branching degree, with architectures ranging from linear homopolymer to star bottlebrushes, and observed that the hydrodynamic radius, *T*_c_, and radial growth rate decreased and the nucleation density increased with increasing branching degree.^16^ Bersenev et al. reported that semicrystalline molecular bottlebrushes form banded spherulites, with the noncrystalline backbones being expelled toward the interlamellar amorphous gaps.^17^ Interesting superstructure packing was recently reported in bottlebrush poly(*n*-alkyl methacrylate)s.^18^ Crystallization and chain packing were systematically investigated in a series of syndiotactic α-olefin mBBs.^19,20^ In these two later cases, the side chain lengths are short, typically less than 22 carbon atoms.

The strong tethering effect and the associated steric hindrance are critical to mBB crystallization. Varying the side chain grafting density (σ) allows for systematically investigating the effects. We recently demonstrated that this tethering effect led to the formation of mBB crystalsomes.^21^ Crystalsomes are a class of hollow polymer nanocrystals with broken translational symmetry, which was developed originally using nanoemulsion solution crystallization.^22−33^ A recent study showed that similar crystalsome structures can be formed by cocrystallizing end-functionalized polymers with nanoparticles.^30^ It was also shown that the radius of curvature of crystalsomes can be tuned by both the crystal thickness and the mBB grafting density.

In this work, the effect of grafting density on mBB crystallization behavior in bulk is investigated for a series of mBBs with poly(ethylene oxide) (PEO) side chains grafted to a methacrylate backbone polymer. Differential scanning calorimetry (DSC) was utilized to ascertain the grafting density effect on crystallization using nonisothermal, isothermal, and self-seeding methods. The crystalline structure and semicrystalline morphology were studied using wide-angle X-ray diffraction (WAXD), small-angle X-ray scattering (SAXS), and Fourier transform infrared spectroscopy (FTIR). The morphology of mBB spherulites was investigated through in situ polarized light microscopy (PLM) with hot stage, scanning electron microscopy (SEM), and atomic force microscopy (AFM). Our results demonstrate that the side chain grafting density significantly influences the nucleation and growth process of mBB crystals in bulk.

## Experimental Section

### Materials

CuCl (97%, Alfa Aesar) was purified by stirring in a vial with glacial acetic acid (certified ACS, Fisher Scientific). The solid was vacuum filtered and washed multiple times with absolute ethanol and inhibitor-free diethyl ether sequentially. The purified CuCl was collected into a vial and dried under a high vacuum to remove residual solvent and stored in a desiccator. *N*,*N*,*N*′,*N*″,*N*″-Pentamethyldiethylenetriamine (99%, Acros) was stirred for several hours over calcium hydride at room temperature and purified by vacuum distillation prior to use. Dry tetrahydrofuran was distilled over sodium benzophenone ketyl and used immediately. Linear PEO (*M*_n_ = 30 kDa, *D̵* = 1.01) was purchased from Polymer Source Inc. and kept in a desiccator at 0 °C before use. All other chemicals used in this work were purchased from Fisher Scientific or Aldrich and used without further purification.

The synthesis of linear mBBs bearing PEO side chains was performed using a copper(I)-catalyzed azide–alkyne cycloaddition (CuAAC) process (Figures S1–S3 in the Supporting Information).^34−36^ The backbone polymer contains spacer groups that enable higher grafting densities of mBBs using the grafting-to approach (see Supporting Information for details). Table 1 lists the molecular characteristics of the mBB polymers, including the number-average molecular weight (*M*_n_) and dispersity (*D̵*) as determined by size exclusion chromatography (SEC) analysis with respect to linear polystyrene standards. A PL GPC-50 Plus system with Agilent Mixed-B or GRAL columns was used, and *N*,*N*-dimethylformamide containing 50 mM LiBr was employed as the mobile phase. The grafting density σ was calculated using the molar ratio of the backbone repeat units to the side chain polymer in the feed and the ratio of peak areas of the brushes and unreacted side chains from SEC analysis of the final reaction mixture. Detailed synthesis and chemical structures of the mBB samples can be found in the Supporting Information. The samples are abbreviated as mBB-*x*, where *x* is the percent PEO side chain grafting density. In a 100% grafting density mBB, there would be one PEO side chain for every other backbone carbon atom, and it would be denoted as mBB-100.

**Table 1:** Molecular Characteristics of the Synthesized PEO-mBB Polymers

mBB sample	*M*_n,SEC_ (Da)	*D̵*	grafting density σ (%)^a^	*w*^b^
mBB-19	6.82 × 10^5^	1.15	19.1	0.80
mBB-47	9.10 × 10^5^	1.17	46.9	0.91
mBB-61	1.14 × 10^6^	1.14	61.1	0.93
mBB-73	1.12 × 10^6^	1.14	72.5	0.94
mBB-94	1.20 × 10^6^	1.13	93.9	0.95

a Grafting density calculated from the ratio of the peak areas of the brushes and unreacted side chains from SEC analysis of the final reaction mixture and the amounts of the backbone polymer and PEO in the feed.

b Mass percentage of grafted PEO in each mBB calculated by , where *m*_PEOside chain_ and *m*_total_ are the molar mass of all the PEO side chains in one mBB molecule and the total molar mass of the mBB, respectively. DP_BB_ is the backbone degree of polymerization and σ is the grafting density.

### Characterization

#### Thermal Characterization

DSC measurements were conducted via a TA Instruments DSC Q2000 using aluminum Tzero pans for isothermal and nonisothermal experiments under N_2_ atmosphere. Nonisothermal heat–cool–heat temperature profiles were used to assess bulk crystallization behavior of mBBs. An isothermal protocol was used to develop the time-evolution of crystallinity at a given *T*_c_. For isothermal experiments, the sample was first held at 100 °C for 10 min to erase the thermal history and then quenched to *T*_c_. Isothermal temperatures were held for 1 h to allow the chains to fully crystallize before being remelted at 10 °C/min to study the melting behavior.

To assess the grafting density effect on the retention of chain conformation upon crystal melting, a self-seeding protocol was employed. First, the mBB samples were heated to 100 °C at 10 °C/min and held for 10 min to erase the previous thermal history, followed by cooling to 0 °C at 10 °C/min to crystallize the sample from the isotropic melt. The sample was then reheated to a seeding temperature, *T*_ss_, at 10 °C/min. After the seeding step, the sample was cooled to 20 °C at 10 °C/min to crystallize and subsequently heated to another *T*_ss_. The corresponding onset and peak crystallization temperatures were monitored as a measure of the memory retention in the sample.

#### Structure Characterization

Simultaneous SAXS and WAXD experiments were performed on the Xenocs DEXS system with 1 M Pilatus (SAXS) and 100 K Dectris (WAXD) detectors with 600 s acquisition time in vacuum. The sample-detector distances were calibrated with silver behemate. mBB samples were melted at 100 °C for 10 min in clean aluminum foil, then cooled at 0.5 °C/min to 25 °C. Samples were mounted using scotch tape such that the flat surface produced by melting and crystallization was clear of the tape and in the same plane as the standards used for calibration.

#### FTIR Spectroscopy

FTIR spectra were collected on a Bruker Invenio-R spectrometer equipped with a Linkam instrument HFS-350 hot stage in a custom-built mount. Scans were taken at 2 cm^–1^ resolution, averaging 32 scans for background and sample collection. Approximately 50 μm thick open-faced mBB films were cast on KBr coverslips (International Crystal Laboratories #0000-7093) from 500 μL of 1 mg/mL chloroform solutions, and dried in vacuum for at least 2 days before measuring. Five kDa alkyne-terminated PEO (A-PEO_114_) samples were melt-pressed between KBr coverslips due to their low viscosity. To eliminate processing history, FTIR samples were heated to 100 °C on the hot stage for 10 min, cooled at 10 °C/min to 21 °C, and allowed to crystallize overnight in a vacuum chamber before measuring. The cooling rate was maintained through the full temperature range by blowing N_2_ through the cooling loop in the hot stage. Before measurement, a scan at 30 °C was taken, and then the temperature ramped at 10 °C/min to 70 °C. Melt state scans were taken after a 20 min isothermal hold at 70 °C. Spectra were normalized to the C=O stretching peak at 1735 cm^–1^. To account for the different C=O to PEO ratios in different samples, normalized mBB spectra were multiplied by (2 + σ) for mBB and the control sample (see Figure S1 for the C=O content in mBBs). A-PEO_114_ contains one C=O per chain, and its spectrum was multiplied by 1.

#### Morphology Characterization

MBB films were melted on glass slides before assessing spherulite morphology. The glass slides were washed with deionized H_2_O and isopropyl alcohol and stored in isopropyl alcohol until needed. To prepare the mBB films, 0.3 mg of an mBB powder sample was deposited onto a preheated glass slide at 100 °C on a Linkam T95-PE hot stage. The sample was allowed to fully melt for 5 min. Afterward, the film was flattened via sandwiching with another glass cover slide at 100 °C. The sample was then cooled to an isothermal temperature, and the spherulite growth was observed in situ via an Olympus BX51 microscope equipped with cross polarizers.

SEM samples were prepared using a similar method. The mBBs for observation of spherulites under SEM were first heated to 100 °C, pressed with a PTFE plate to create an open-faced film, melted for another 10 min to erase thermal history, and then allowed to crystallize isothermally for 24 h at preset temperatures. SEM characterization was performed on a Zeiss Supra 50VP scanning electron microscope. Samples were sputtered with a thin layer of platinum using a Cressington 208HR sputter coater. AFM imaging was conducted with a Bruker Multimode 8 in QNM mode with Scanasyst-air silicon nitride triangular probes.

## Results and Discussions

### Grafting Density Effect on Nonisothermal Crystallization of PEO mBBs and the Equilibrium Melting Temperature

**Figure 1:**
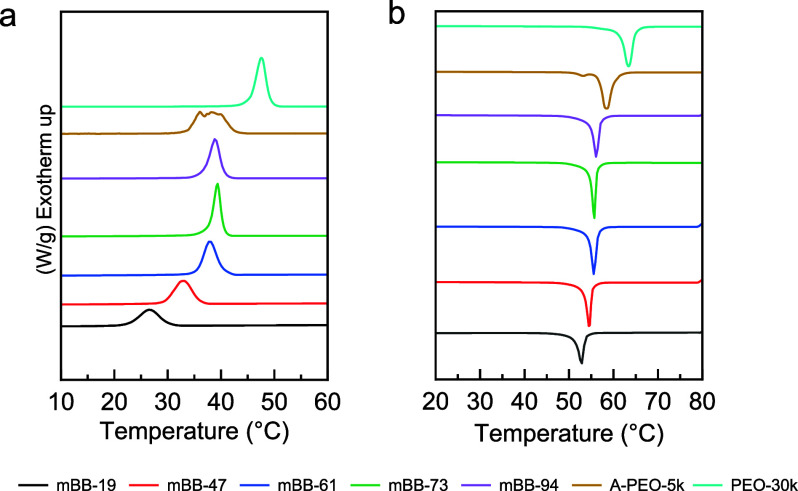
DSC first cooling (a) and second heating (b) thermal traces of mBB and PEO samples.

**Figure 2:**
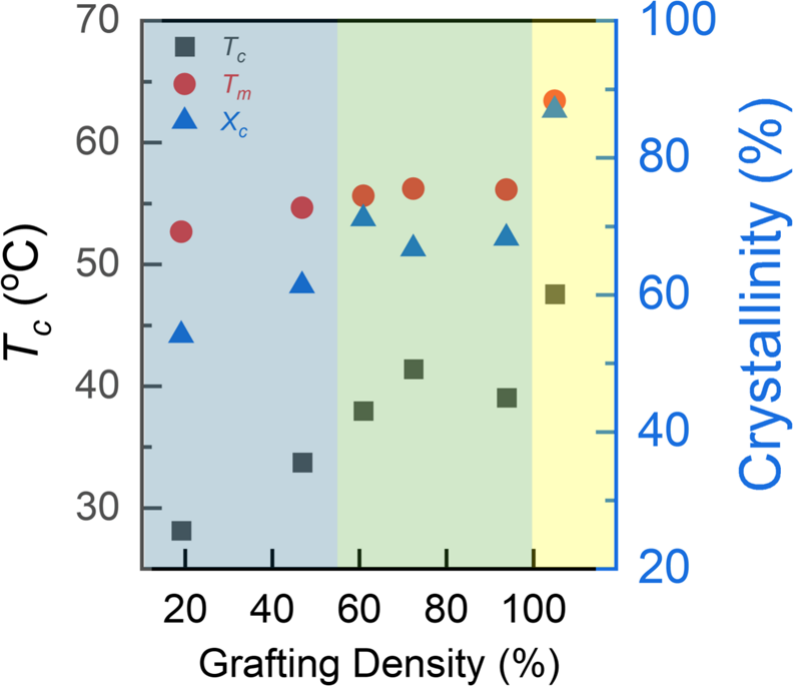
Summary of the nonisothermal crystallization results of mBB-*x*. The yellow region on the right is from the *l*-PEO_682_ control.

Nonisothermal DSC experiments were performed on the mBBs to determine their bulk crystallization and melting behaviors. Figure 1 presents the first cooling and second heating thermograms, and Table 2 lists the representative thermal transition temperatures and crystallinities of the sample from the second heating thermograms. A-PEO_114_, the side chain polymer before coupling onto the backbone, was first used as the control. Multiple exothermic crystallization peaks are observed upon cooling, which can be attributed to the low molar mass and the end-group effect.^37^ We also used a higher molar mass, 30 kDa, linear PEO (*l*-PEO_682_), as the second control sample. In this case, a single exothermic peak, with a *T*_c_ of 47.57 °C, was seen without an apparent chain end effect. For all the mBB samples, a lower *T*_c_, *T*_m_, and crystallinity (*X*_C_) were observed, with a decrease in *T*_c_ of 19.45 °C between *l*-PEO_682_ and mBB-19. The grafting density effects on the thermal transition are evident: as the grafting density increases, *T*_c_ rises from 28.12 °C for mBB-19 to 39.04 °C for mBB-94, a 10.92 °C increase. mBB-61, -73, and -94 have relatively similar *T*_c_ (38–39 °C), and the crystallization exotherms are narrow, while the *T*_c_ values of mBB-19 and mBB-47 are significantly lower, with a broader exotherm. The onset temperatures of the crystallization peaks show a similar trend. With increasing the grafting density, both the crystallization peak and onset temperatures reach a maximum with mBB-73 and then slightly decrease when further increasing the grafting density to 93.9% for mBB-94. For heating, the *T*_m_ depression is less significant, with a 10.77 °C drop for mBB-19 from the *l*-PEO_682_ control. Again, the five mBBs can be separated into two groups, where mBB-61, -73, and -94 have higher *T*_m_, while mBB-19 and -47 show slightly lower transition temperatures. The *T*_c_ and *T*_m_ trends are plotted in Figure 2.

**Table 2:** Non-isothermal Crystallization Data for mBB Polymers

sample	*T*_c_, peak (°C)^a^	*T*_c_, onset (°C)^a^	*H*_c_ (J/g)^a^	*T*_m_, peak (°C)^a^	*T*_m_, onset (°C)^a^	*H*_f_ (J/g)^a^	*X*_c_ (%)^a^	*X*_C_^N^ (%)^b^
mBB-19	28.12	31.87	88.4	52.66	51.64	84.9	43.2	54.0
mBB-47	33.72	36.12	109.8	54.64	53.55	109.04	55.6	61.3
mBB-61	37.95	40.37	131.2	55.62	54.57	129.6	65.9	71.1
mBB-73	41.38	42.39	131.0	56.2	55.38	123.0	62.6	66.7
mBB-94	39.04	40.7	126.3	56.13	55.02	127.7	65.0	68.3
A-PEO-5k	41.48	45.33	168.6	59.85	51.38	173.8	88.4	88.4
PEO-30k	47.57	49.26	167.2	63.43	61.55	172.5	87.7	87.7

a Determined from DSC.

b Normalized crystallinity to the mass percentage of PEO side chains in the mBB.

The polymer crystallinity was derived from the heat of fusion from the second heating thermograms. Compared with the *l*-PEO_682_ control (crystallinity *X*_c_ = 87.7%), mBBs show a much lower crystallinity, with the lowest value of 43.2% observed in mBB-19. A normalized crystallinity was calculated following , where *X*_C_^N^ and *w* are normalized crystallinity and the mass percentage of the PEO side chains in an mBB, respectively. Using *X*_C_^N^ for discussion removes the mass fraction of the backbone, which cannot crystallize and varies with grafting density. The crystallinity slightly increases after normalization, with the lowest value of 54% for mBB-19, which is still significantly lower than that of the *l*-PEO_682_ control. We again see the behavior of two groups of samples with mBB-61, -73, and -94 having similar *X*_C_^N^ values, while mBB-19 and -47 are much less crystalline.

When comparing the crystallization behaviors of mBBs with their linear counterparts, three factors are important: (1) noncrystalline moiety contents. While a linear semicrystalline chain could potentially be fully crystalline, the mBB’s backbone and spacer are intrinsically noncrystalline and are excluded from the crystal lattice during crystallization. In the present case, the crystallizable PEO mass content *w* in mBBs is 80% for mBB-19 and 95% for the highest grafting density mBB-94, leaving 20 and 5% noncrystalline moieties, respectively (Table 1). (2) The tethering effect and intra-mBB packing. A direct consequence of the side chain tethering is that intra-mBB packing (that is, packing of side chains in the same mBB molecule) must be an integral part of mBB crystallization, and this process could be kinetically hindered when the side chains become more crowded with a high grafting density. (3) The tethering effect and inter-mBB packing. Since the side chains are tethered to the backbone, their freedom of diffusion is limited. The mBB molecules, consisting of hundreds of side chains, must collectively diffuse to the crystallization growth front in order to attach to a crystal. This framework can guide our understanding of the observed nonisothermal crystallization data. As the grafting density increases, *T*_c_ significantly rises. This can be attributed to the likely increased side chain alignment in higher grafting density samples, which facilitates nucleation, a point we will return to in the self-nucleation study. mBB-19 has the lowest grafting density, containing ∼20% of noncrystallizable moieties, which is the major reason for its low *T*_c_, *T*_m_, and crystallinity. The crystallization exotherm is also broader for mBBs with relatively low grafting densities, suggesting that the crystallization kinetics is slowed down in the low grafting density samples as the noncrystallizable groups are expelled from the side chain crystals during crystallization. The increasing normalized crystallinity with increasing grafting density indicates that the noncrystallizable component hinders crystal packing, which is consistent with the melting point trend. Similar observations have been reported for semicrystalline block copolymers or random copolymers.^38,39^

**Figure 3:**
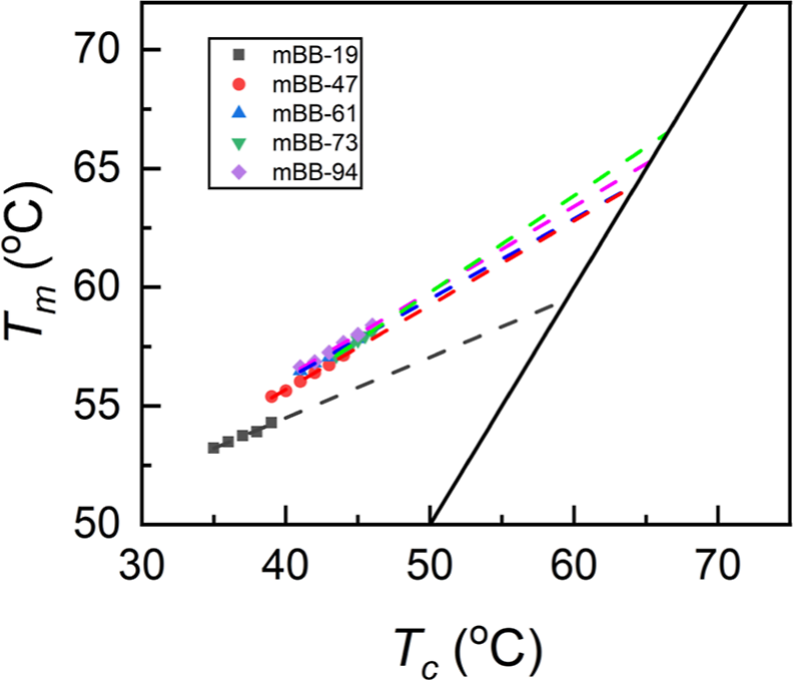
Equilibrium melting temperatures of PEO molecular bottlebrush samples with different grafting densities.

**Figure 4:**
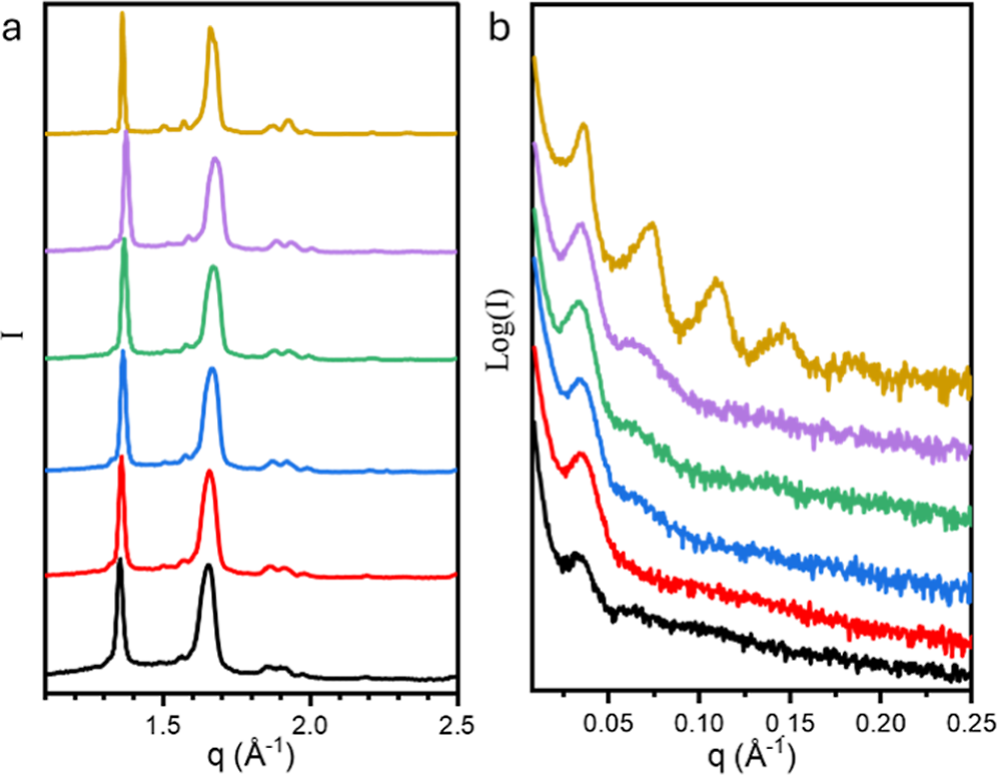
(a) WAXD and (b) SAXS of mBB and A-PEO_114_ samples at 21 °C. From top to bottom, A-PEO114, mBB-94, 73, -61, 47, 19-2.

The effect of noncrystallizable contents can be better revealed by estimating the equilibrium melting point of the mBB crystals, which removes the kinetic factor. The equilibrium melting temperature of polymer crystals, *T*_m_^0^, is defined as the melting point of infinitely large extended chain crystals and is typically used as the reference point to estimate undercooling during crystallization.^40^ Several methods have been used to estimate *T*_m_^0^. In this study, the classical linear Hoffman–Weeks method is employed, and the result is shown in Figure 3.^40,41^*T*_m_^0^ was estimated by the intercept of the *T*_m_ vs *T*_c_ line with the equilibrium line. Based on the *T*_m_^0^, the samples can be categorized into two groups: *T*_m_^0^ of 59.5 °C for mBB-19 and 64.3–66.5 °C for mBBs with grafting densities of 47–94% (Tables S1 and S2). *T*_m_^0^ of 64.3–66.5 °C is consistent with the reported low molar mass PEO,^37,42^ while mBB-19 is 5 °C lower than other mBBs. For mBB crystals, an increase in *T*_m_ with *T*_c_ suggests that the mBB crystals thicken with increasing *T*_c_. To this end, WAXD and SAXS experiments were conducted, and the results are shown in Figure 4. The samples were melted at 100 °C for 10 min in clean aluminum foil, then cooled at 0.5 °C/min to 25 °C. WAXD confirmed the monoclinic structure of PEO crystal (Figure 4a) while SAXS patterns showed typically 2D lamella scattering (Figure 4b). The long period of the lamellae can be calculated to be 19.4, 18.5, 18.8, 18.5, 17.6, and 17.3 nm for mBB-19-2 (a second mBB-19 with a grafting density of 19.4%, see Supporting Information for more information), -47, -61, -73, -94, and A-PEO_114_. Considering the 7_2_ helical conformation of the PEO chain in the monoclinic unit cell of *a* = 0.805 nm, *b* = 1.304 nm, and *c* = 1.948 nm,^43^ the PEO side chain would have an extended chain conformation of 31.7 nm. This confirms that PEO chains in the mBBs are indeed folded, consistent with the Hoffman–Weeks results. Note that for the lower grafting densities, the noncrystallizable moieties can be viewed as uniformly distributed defects in the theoretically infinitely large extended chain crystals, significantly depressing the melting pointing of the latter. Comparing mBB-19 with the rest of the mBBs, higher grafting densities reduce the defect concentration and, therefore, increase *T*_m_^0^.

### Grafting Density Effect in Isothermal Crystallization of mBBs

**Figure 5:**
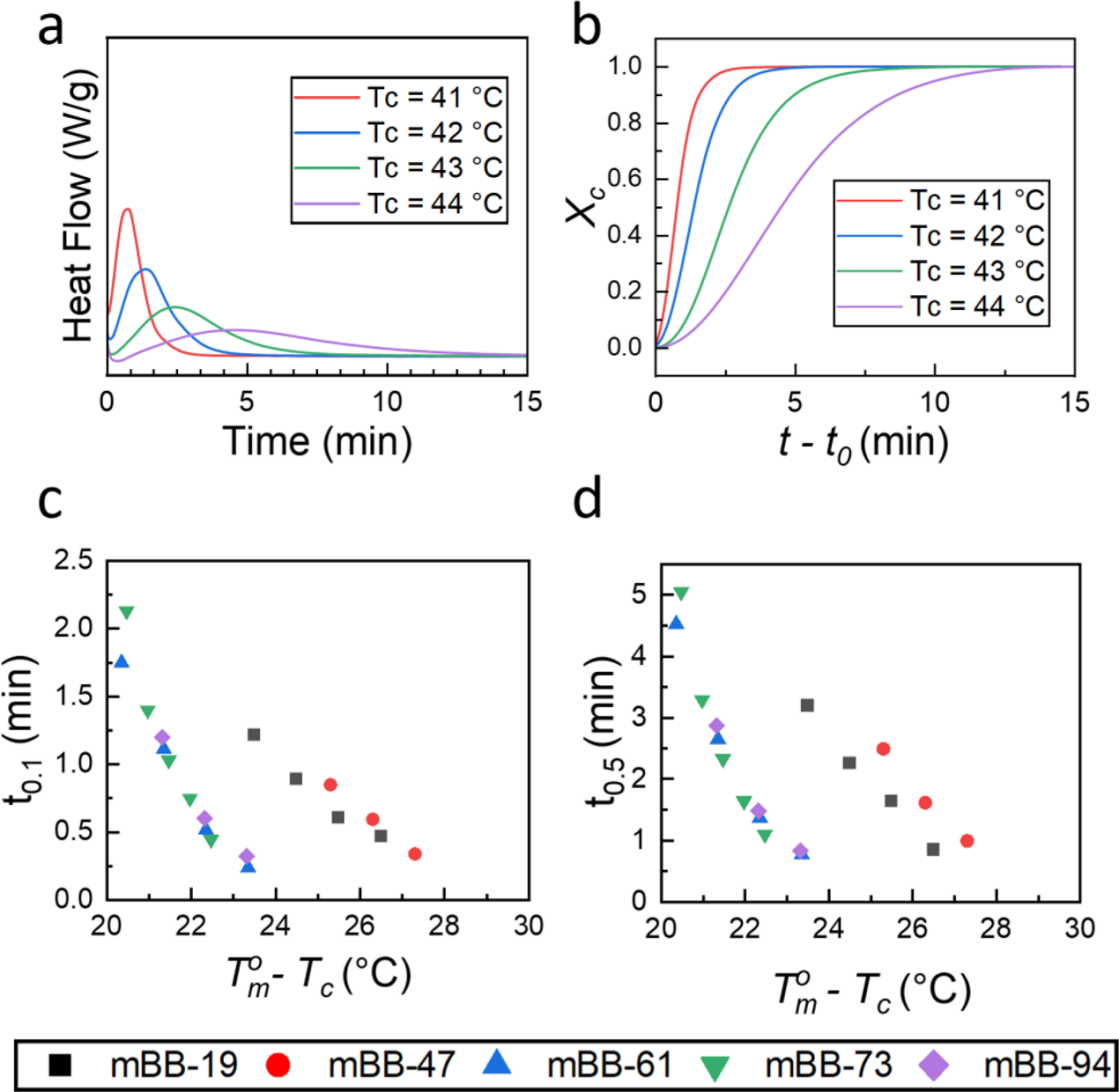
Isothermal crystallization of mBB-*x*. (a,b) Isothermal crystallization exotherms as a function of time at different temperatures (a) and evolution of crystallinity as a function of crystallization time starting from the onset of crystallization (b) for mBB-61. (c,d) *t*_0.1_ (c) and *t*_0.5_ (d) of mBBs vs undercooling (*T*_m_^0^ – *T*_c_).

The isothermal crystallization experiments were conducted using DSC. Figure 5a shows the representative isothermal exotherms for mBB-61. The rest of the results can be found in the Supporting Information (Figure S4). The crystallization exotherm is broader at higher *T*_c_ as the crystallization time is increased at lower undercooling (*T*_m_^0^ – *T*_c_). The exotherm sharpens and shifts toward shorter crystallization times at higher undercoolings. Parameters *t*_0.1_ and *t*_0.5_ (Figure 5c,d), representing the time to reach 10 and 50% crystallinity, are estimated based on the development of relative crystallinity (Figures 5b and S5). Note that due to the different crystallization kinetics, meaningful data can be obtained for mBBs at different undercooling ranges. Both *t*_0.1_ and *t*_0.5_ decrease with increasing the undercooling, highlighting the accelerated crystallization processes. The results also reveal that, for certain grafting densities, similar kinetics are observed, where mBB-61, -73, and -94 collapse to one master curve while mBB-19 and -47 to another. The overall trend is similar to the nonisothermal results and confirms two regimes for lower and higher grafting density samples. The higher grafting density samples (mBB-61-94) exhibit significantly shorter *t*_0.1_ and *t*_0.5_ than lower grafting density mBBs (mBB-19-47), indicating that nucleation and growth are much faster in mBB-61-94, in accordance with the higher *T*_c_ observed in these samples in nonisothermal experiments. The sluggish nucleation kinetics in mBB-19 and -47 can be attributed to the relatively high noncrystallizable moiety contents compared to their higher-grafted counterparts. The exclusion of the relatively large number of these defects from the crystalline domain slows down the crystallization kinetics.

Avrami analysis was used to understand the isothermal crystallization process of mBBs, *X*(*t*) = 1 – exp(−*Kt*^*n*^), where *n* is the Avrami exponent and *K* is the kinetic parameter(Figure S6).^44^Figure S7 summarizes the overall Avrami crystallization kinetics. The overall trend for *K* increases with increasing supercooling, highlighting the accelerated crystallization process as the crystallization temperature decreases. Two regimes of the behavior of mBB-19-47 and mBB-61-94 are again seen, consistent with the nonisothermal and *t*_0.1_ and *t*_0.5_ results. The Avrami exponent ranges from 1.5 to 2.2, consistent with the literature values for bottlebrush PEO side chains.^15,16^ For homopolymer PEO, the range of *n* values is reported to be between 1.4 and 4, increasing with molecular weight.^45^ The Avrami exponent for mBBs is generally lower, which can be attributed to the tethered side chains to the backbone, imposing an intrinsic 1D nature of the mBB backbone to the crystal growth and reducing *n*. Similar observations were reported for PEO chains tethered to silica nanoparticles, exhibiting a nucleation-dominant behavior and Avrami values of lower than 1.^46^ This behavior is also similar to the confined crystallization of PEO, which has been extensively studied.^32,46−48^

### mBB Crystal Morphology

**Figure 6:**
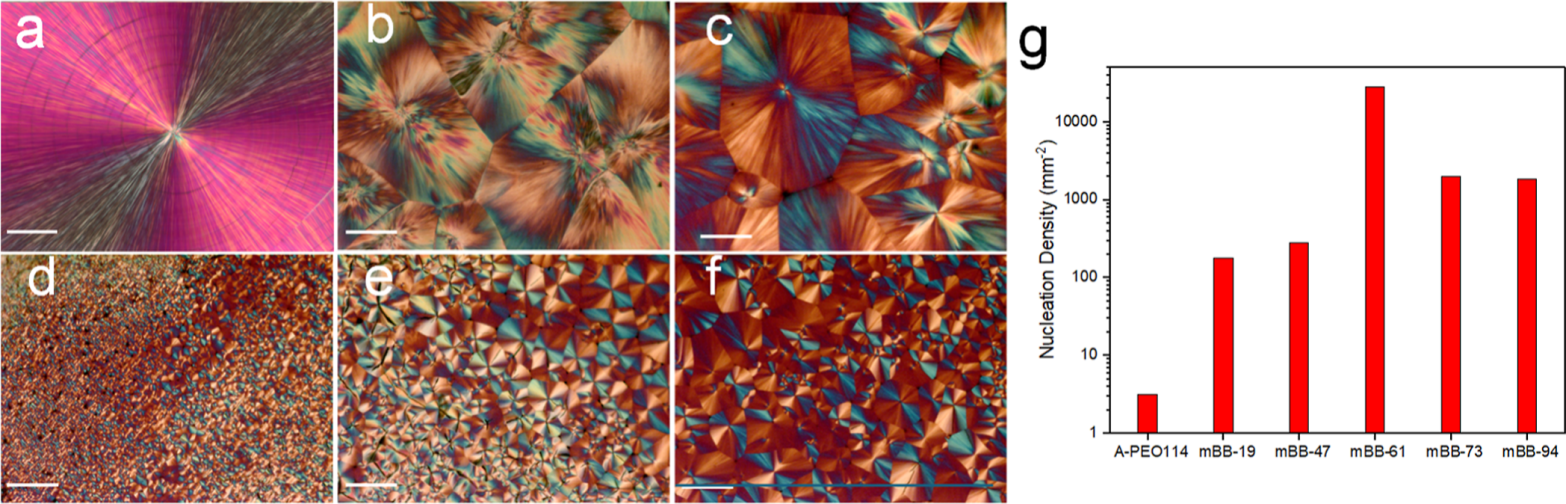
PLM images of spherulites crystallized at 44 °C for 12 h. The nucleation density of A-PEO_114_ at 44 °C was too low, and the sample was quenched from 100 to 25 °C to increase nucleation. (a) A-PEO_114_, (b) mBB-19, (c) mBB-47, (d) mBB-61, (e) mBB-73, and (f) mBB-94. (g) Nucleation density of pressed spherulites (mm^–2^). Scale bars are 40 μm.

Solution-grown mBB crystals were studied previously.^21^ Instead of flat crystals, hollow spherical crystalsomes were observed, which was attributed to the bilayer structure of the lamellae consisting of crystalline stems and noncrystallizable moieties. The diameter of the crystalsomes decreased with increasing the grafting density.^21^ In this work, isothermal crystallization of thick films was conducted to study mBB spherulite morphology using a combination of PLM, SEM, and AFM experiments. The PLM micrographs of mBB spherulites are shown in Figure 6. After isothermal crystallization at *T*_c_ = 44 °C for 12 h, large spherulites are seen for *l*-PEO and mBB-19, while the observed spherulite size gradually decreases for mBB-47 and -61, and slightly increases again for mBB-73 and -94. Spherulites formed under the same undercooling are shown in Figures S8 and S9. Five mBB PLM images were taken at undercooling temperatures of 20 and 25 °C. A consistent trend can be seen that the nucleation density first increases and then decreases with increasing the grafting density. mBB-61 shows a much greater nucleation density, and the nucleation densities of mBB-47 and -73 are similar, as are mBB-19 and -94. The bell-shaped nucleation behavior shown in Figure 6g can also be explained based on the previously discussed three factors, namely, the noncrystallizable moiety content, the tethering effect/intra-mBB packing, and the tethering effect/inter-mBB packing. The low nucleation density of the lower grafting density mBBs can be attributed to the relatively high noncrystallizable moiety content—they must be excluded from the crystalline domain during nucleation, a process that slows down the nucleation rate. The observed nucleation density, therefore, first increases with the side chain grafting density. However, in the very high grafting density regime, intra-mBB packing of the PEO side chains becomes increasingly more difficult due to overcrowding. Moreover, the adjacent mBB molecules must also adjust local orientation to facilitate nucleation. Both factors could lead to the fall of nucleation density at high grafting densities.

**Figure 7:**
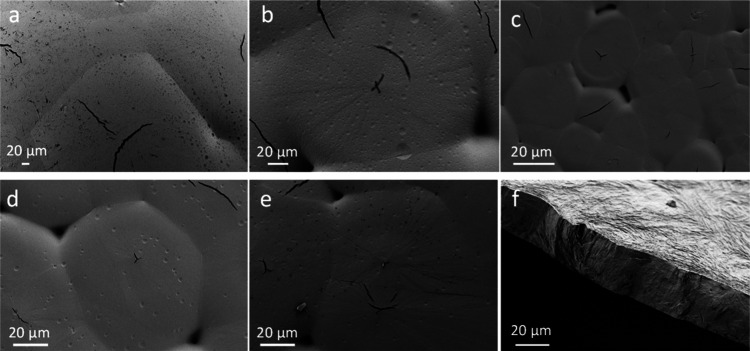
SEM images of pressed mBB spherulites grown at *T*_c_ = 44 °C. (a) mBB-19, (b) mBB-47, (c) mBB-61, (d) mBB-73, and (e) mBB-94. (f) A SEM image highlights a cross-section of the mBB-94 sample.

mBB spherulites were imaged using SEM (Figure 7), where cracks are seen in all the spherulites.^49−52^ While some are tangentially oriented to the spherulites, many others are arbitrarily orientated with respect to the radial directions of the spherulites. The formation of the cracks is likely due to the lack of entanglements between mBB molecules, and the lamellae are, therefore, relatively easily delaminated. We notice that there tend to be “holes” at the impingement points where three or more spherulites meet, likely due to the depletion of polymers at these junctions, and again, this observation reflects the low entanglement of the bottlebrushes and is likely associated with the large molecular size. Additional evidence of low entanglement is evident in Figure 7f, which shows the cross-sectional image of mBB-94 spherulites and the clear fracture surface, suggesting the brittle nature of the spherulites.

**Figure 8:**
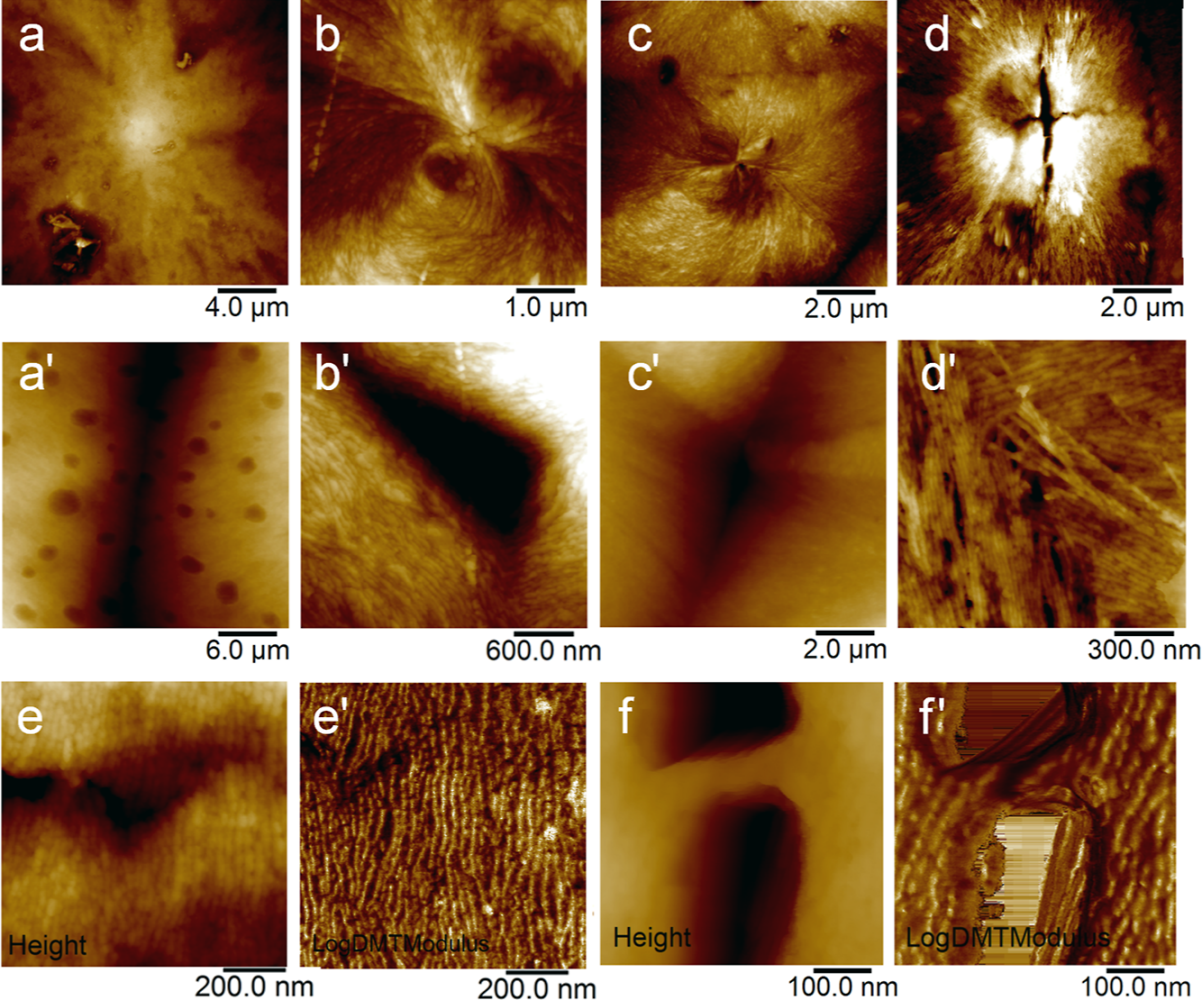
AFM topography scans of mBB spherulites grown at *T*_c_ = 44 °C. (a) mBB-19, (b) mBB-61, (c) mBB-73, and (d) mBB-94. (a′–d′) Height images show the interfacial region between two or more spherulites of mBB-19, mBB-61, mBB-73, and mBB-94, respectively. Higher magnification of an mBB-94 spherulite was recorded along two directions in the spherulite: (e,e′) the horizontal direction and (f,f′) the vertical direction. (e',f′) show the log DMT modulus images.

Further analysis of the mBB spherulites is provided by AFM topography scans, as shown in Figure 8. Spherulites shown under AFM are observed to have lamellae oriented from the nucleation site growing radially outward. For higher grafting density mBBs, the edge-on lamellae are apparent from the height topography. For mBB-19, the edge-on lamellae are not evident, likely due to the high content of the noncrystallizable moieties obscuring the lamellar structure. Lamellar branching near the center of the spherulites is particularly clear from Figure 8b,c. Films remained open-faced during the cooling process to acquire the images of both the SEM and AFM samples. Given the PEO’s affinity for water, some slight condensation may have partially dissolved the surface of the spherulites, seen in the SEM (Figure 7b,d,e) and the AFM images (Figure 8a,a′)—like the well-known breath figure effect.^53^ At the intersection of two or three spherulites, an impingement area can be seen with the material depletion from the molten film during crystallization, consistent with the SEM results (Figure 8 second row). The relative orientation of the lamellae in adjacent spherulites varies, e.g., ∼110° in Figure 8b′ bottom and ∼60° in Figure 8d′, which is due to the relative locations of the spherulite center and the impingement points. Of interest is that, in Figure 8d′, the edge-on lamellae propagate and grow into the domain of the adjacent spherulite, suggesting a poorly defined growth front. Sharp bending of a few edge-on lamellae can also be seen in the figure, and the bending bridges the adjacent spherulite. This suggests that a single mBB molecule could span across the two spherulites, each end may have started crystallization in its own spherulite domain independently, and the crystal growth propagates to the mBB chain center, leading to the bent shape edge-on crystals. This observation therefore provides a good view of the perspective of the size of mBB molecules and the individual lamella crystals.

Figure 8d shows a large crack in the center of the mBB-94 spherulites. The crack then propagates both horizontally and vertically. Two crack regions were selected for high-magnification imaging. In Figure 8e,e′, the height and modulus images reveal that the lamellae are orientated nearly orthogonal to the crack, providing a bridging effect. This leads to a relatively small crack opening, similar to the crazing effect. On the other hand, the height and modulus images in Figure 8f,f′ show that the lamella is nearly parallel to the crack surface; a large opening is therefore formed. A few thin chain fibrils bridge the two crack open surfaces. The modulus image in Figure 8f′ reveals that the lamella was pulled and became oblique to the crack surface.

**Figure 9:**
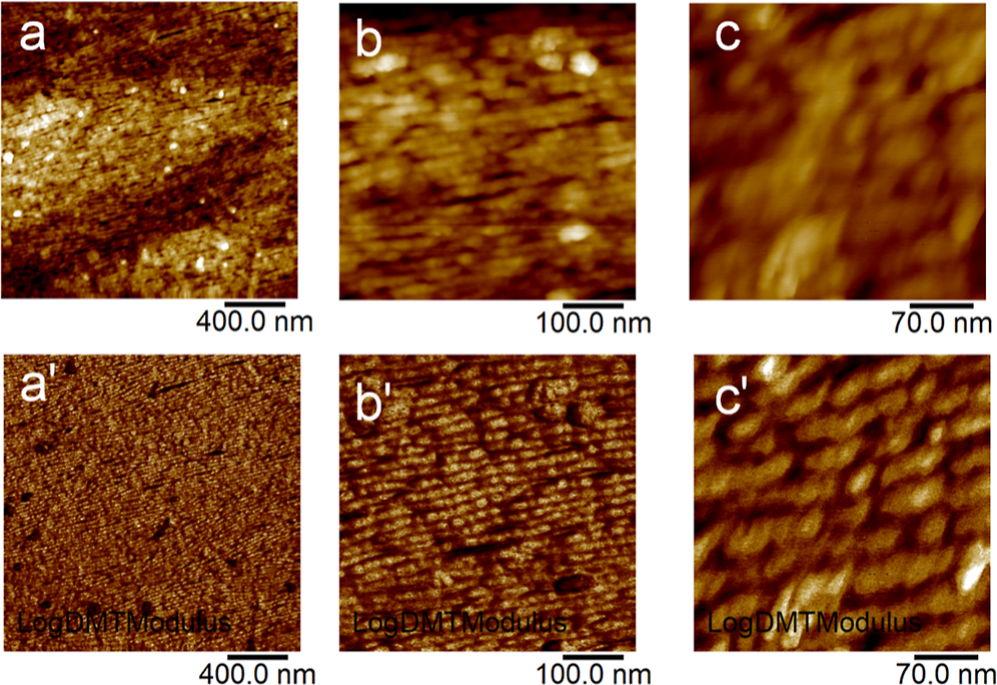
High-resolution AFM scans of mBB-94 spherulites grown at *T*_c_ = 44 °C. (a–c) Show the height images with increasing magnification, and (a′–c′) are the corresponding modulus images.

High-magnification AFM scans were conducted to investigate the edge-on lamellae in mBB-94 (Figure 9). Relatively uniform orientation of the crystals is evident. Additional contrast can be seen through the observation of the modulus map, which was acquired using the Derjaguin–Muller–Toporov (DMT) model through nanomechanical feedback from tip–sample interactions during the scan.^54,55^ The apparent lamellar period was determined to be 21.5 ± 0.8 nm using FFT analysis of the high-frequency data points acquired from the log DMT modulus image (Figure S10), consistent with the SAXS results. Higher magnification scans in Figure 9b,b′ and c,c′ suggest that, along the lamellar direction, the crystals are less smooth at the length scale of ∼30–70 nm and appear segmented. The segmented lamellar appearance might be related to the mBB architecture. The mBB molecular chain length is ∼100–200 nm; given that they could occupy one or a few layers of the lamellae, the continuity of the crystalline layer could be disrupted when multiple mBB molecules merge. More detailed studies will be conducted to understand this growth process.

### Grafting Density Effect on mBB Crystal Melting

**Figure 10:**
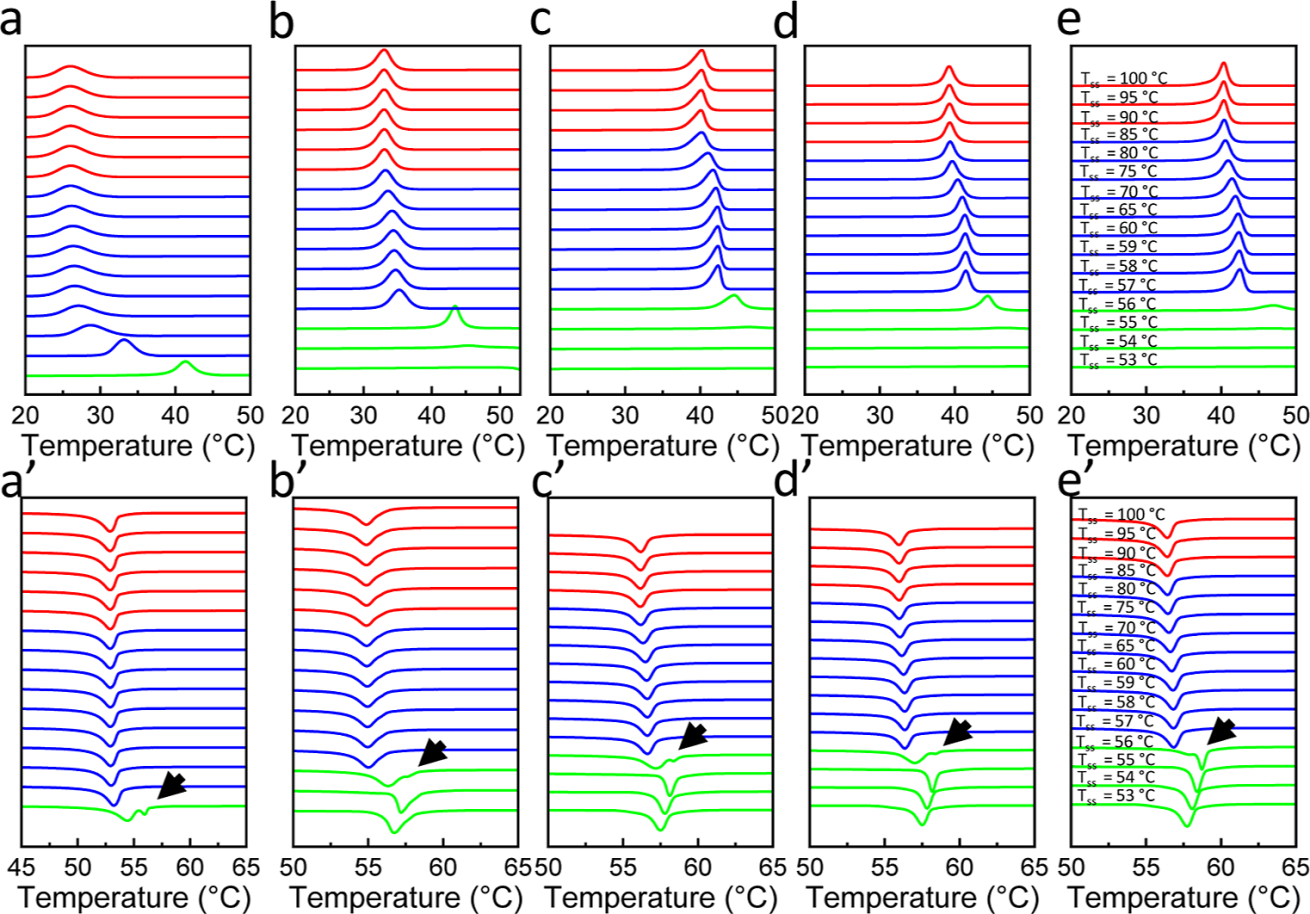
DSC crystallization and melting thermograms of mBBs seeded at *T*_ss_. (a–e) Crystallization exotherms after cooling from *T*_ss_ for (a) mBB-19, (b) mBB-47, (c) mBB-61, (d) mBB-73, and (e) mBB-94 samples. The corresponding melting endotherms are shown on the bottom row for (a′) mBB-19, (b′) mBB-47, (c′) mBB-61, (d′) mBB-73, and (e′) mBB-94 samples. The black arrows denote the locations of double melting peaks and the end of domain III, shown in green, transitioning to the self-seeding domain II, shown in blue. The red traces show the point at which memory is erased and domain I is reached.

Since mBBs with different grafting densities have different degrees of side chain stretching, this brush characteristic could manifest in the crystal melting kinetics. To this end, a self-nucleation study was conducted. The self-seeding process in polymer crystallization has previously been studied through solution-based methods in generating polymer single crystals,^56−60^ in thin films to control the crystal morphology,^61^ and in polymer melt to understand the chain memory effect upon melting.^62−65^ In this work, we hypothesize that, because of the forced chain stretching in brush polymers, mBBs with different grafting densities have different degrees of memory of the crystalline conformation in the nominal melting point region. Figure S11 shows the typical temperature profile as described in the experimental section, and the top row in Figure 10 represents the crystallization thermograms after heating the mBBs to a self-seeding temperature (*T*_ss_), ranging from 53 °C (bottom of the figure) to 100 °C (top of the figure). The bottom row depicts the subsequent heating thermograms. Three regions can be identified based on the crystallization and melting behavior, as shown in the green, blue, and red curves in the figure. When *T*_ss_ is low (green curves), we see a high *T*_c_ and multiple melting peaks in the heating thermogram, which is attributed to the remaining crystals providing an efficient nucleating surface for crystallization and thickening upon heating. This domain is conventionally assigned as domain III (DIII). In the blue region, a gradual down-shift of the crystallization temperature is observed during cooling, and a single melting peak is seen upon heating. This is assigned domain II, attributed to the remaining chain orientation in the melt or small crystallites. The red domain in the figure is assigned domain I (DI), where the crystal memory, i.e., the chain orientation, is completely lost.

**Figure 11:**
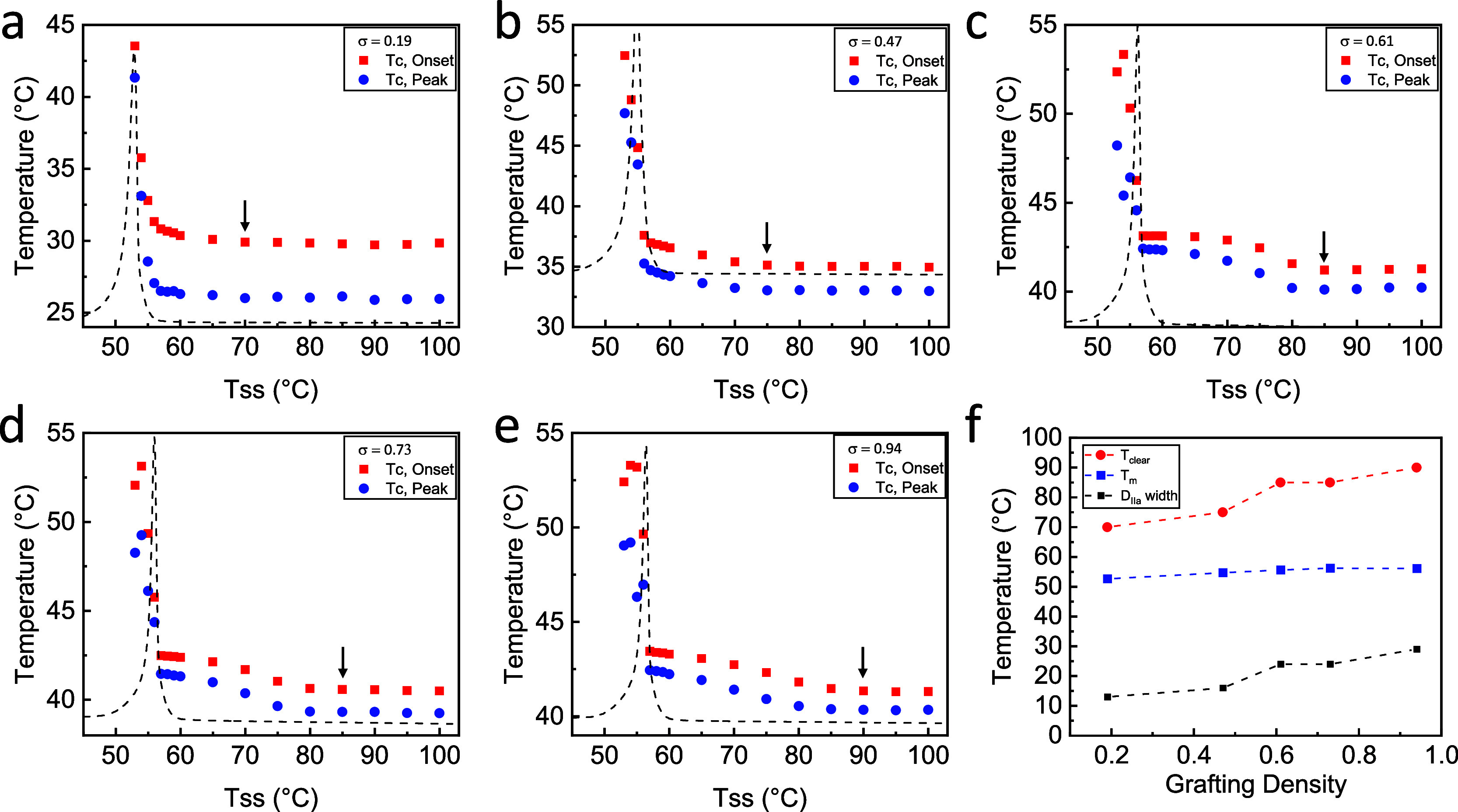
*T*_c_ vs *T*_ss_ plots of molecular bottlebrushes: (a) mBB-19, (b) mBB-47, (c) mBB-61, (d) mBB-73, and (e) mBB-94. Red shows the onset crystallization temperature, and blue shows the peak crystallization temperature. The black arrows indicate the clearing point where the chain memory is erased. The dotted lines are the corresponding second heating thermograms from heat–cool–heat scans. (f) Grafting density dependence of *T*_clear_, *T*_m_, and the width of region DIIa.

*T*_c_ is plotted vs *T*_ss_ to reveal the self-nucleation behavior in mBBs in Figure 11, where the melting thermogram from the second heating of a heat–cool–heat is also included. The trend observed in Figure 11 included a sharp drop in *T*_c_ around the nominal melting point. This captures the transition between DIII to DII, which is also characterized by the establishment and subsequent disappearance of a shoulder in the melting region for a certain seeding temperature (Figure 11). What follows is a gradual decrease in *T*_c_ as *T*_ss_ increases until reaching a constant value. During this time, the PEO side chains are in the self-nucleation domain, which contains small lamellar fragments or chains with residual orientation that serve as nucleation sites for recrystallization upon cooling. The retention of crystalline memory can be assessed by examining the transition temperature between DII and DI, where the polymer enters the isotropic melt state, denoted as the clearing temperature (*T*_clear_) as well as the width of the DIIa region (from the end of endotherm to *T*_clear_). We define the *T*_clear_ as the seeding temperature at which the step change between the crystallization peaks for the two subsequent seeding temperatures is no more than 0.05 °C. From a chain packing perspective, a higher proximity of neighboring chains facilitates more memory preservation. Higher mBB grafting densities encourage the chains to extend orthogonally from the backbone driven by steric repulsion, akin to crystal orientation. The local chains for higher grafting densities preserve more residual memory of the crystalline orientation due to the stretched conformation of the highly grafted brush state. The conformational change of the PEO chain before and after crystallization was monitored by FTIR. While there is no discernible trans conformation difference for mBBs in the melt (Figure S12), the crystals do show a higher trans content in mBBs compared with A-PEO_114_. The increased trans population in mBBs is attributed to the higher amorphous content (lower crystallinity) and suggests that the PEO side chains close to the mBB backbones are more stretched upon crystallization. Figure 11f shows that as the mBB grafting density increases from 0.19 to 0.94, *T*_clear_ rises from 70 to 90 °C, while the melting point only changed by less than 4 °C. The DII width ranges from ∼13 to 29 °C (Table S4), which is broader than most reported linear polymers.^66^ Most recently, molecular weight-dependent melt memory effect in linear PEO was reported.^67^ It was shown that linear PEO with MW > ∼10 kD has an approximately 15 °C wide DIIa. These results, and the reported memory effect studies in linear polymers, further confirm our hypothesis that the memory effect in mBBs is grafting density-dependent, and a higher grafting density leads to a better-preserved memory in mBB crystals upon melting.

## Conclusions

The crystallization and morphology of mBBs bearing semicrystalline PEO side chains with grafting densities from 19.1 to 93.9% were systematically studied using thermal analysis, X-ray, FTIR, and various microscopy techniques. Grafting PEO to a noncrystalline backbone restricts the mobility of the side chains, and the resulting sterically constrained brush PEO has reduced *T*_c_, *T*_m_, and *X*_c_, compared to linear homopolymer PEO. The side chain grafting density imposes a strong influence on the crystallization of mBBs, with a general increase in *T*_c_, *T*_m_, *X*_c_, and *T*_m_^o^ with increasing grafting density, and the increase in *T*_c_, *T*_m_, and *X*_c_ falls off at higher grafting densities. The nucleation density of the spherulites is also grafting density-dependent, with the highest nucleation rate occurring at the intermediate grafting density. The memory effect for brush PEO is strengthened with increasing grafting density, demonstrated by an increase in *T*_clear_, as the higher chain density along the backbone affords more interchain interactions and preserves residual crystalline orientation far past the nominal melting point. The mBB spherulites display brittle fracture behavior due to the lack of chain entanglement in the brush phase. AFM characterization revealed the presence of edge-on crystals with relatively uniform lamellar periods composing the bulk mBB spherulites. The characterization of the architecture effect on the crystallization of mBBs reveals a complex interplay between the side chain packing and the mBB chain structure, ascribed to three major factors: (1) noncrystalline moiety content, (2) the tethering effect and intra-mBB packing, and (3) the tethering effect and inter-mBB packing.

## Supporting Information Available

Synthesis of PEO mBBs with various grafting densities, Avrami analysis, PLM images, AFM images, and the self-nucleation temperature profile (PDF)
