# Supplementary material for: Effect of Grafting Density on the Crystallization Behavior of Molecular Bottlebrushes
Source: Macromolecules. 2024 Aug 20;57(17):8487–97. doi: 10.1021/acs.macromol.4c00752 (PMC11394005; doi:10.1021/acs.macromol.4c00752)
Supplement: Supplementary file 1 — ma4c00752_si_001.pdf [file ma4c00752_si_001.pdf]

Supporting Information

Effect of Grafting Density on the Crystallization Behavior of Molecular Bottlebrushes

Jeffrey T. Wilk,[1§] *Carl T. Furner,*[1§] *Ethan W. Kent,*[2] *Michael T. Kelly,*[2] *Bin Zhao,*[2,*] *and Christopher Y. Li*[1,*]

[1]Department of Materials Science and Engineering, Drexel University, Philadelphia, Pennsylvania 19104, United States

[2]Department of Chemistry, University of Tennessee, Knoxville, Tennessee 37996, United States

[§]*Equal contribution*

* Correspondence to: chrisli@drexel.edu (C.Y.L.); bzhao@utk.edu (B.Z.)

Synthesis of Linear mBBs

Synthesis of linear molecular bottlebrushes bearing PEO side chains with various grafting densities was performed using a copper(I)-catalyzed azide-alkyne cycloaddition (CuAAC) process, as shown in **Figure S1**.[1] The values of number-average molecular weight (M_n) and dispersity ($Đ$) were determined by size exclusion chromatography (SEC) analysis relative to linear polystyrene standards using a PL GPC-50 Plus system with Agilent Mixed-B or GRAL or GRAM columns and *N*,*N*-dimethylformamide (DMF) containing 50 mM LiBr as the eluent. The grafting density was calculated by using the molar ratio of backbone repeat units to the side chain polymer

in the feed and the ratio of peak areas of the brushes and unreacted side chains from SEC analysis of the final reaction mixture.

Alkyne end-functionalized PEO with a molecular weight of 5000 Da was prepared as reported previously. [2-3] The synthesis procedure for the linear functionalized backbone polymer with a degree of polymerization of 801 (PHEMA-N_3-801, Figure S1) used in this work is described in detail in a previous publication.[1, 3] The following is the synthesis procedure of mBB-47 with a backbone DP of 801 and PEO side chains with a DP of 114 (i.e., M_n = 5000 Da). Similar procedures were used in preparing PEO mBBs with different grafting densities. The backbone polymer PHEMA-N_3-801 (10.1 mg, 0.0417 mmol monomer units, delivered from a stock solution in THF) was added to a 7.4 mL vial equipped with a stir bar. THF was evaporated using a nitrogen gas stream. In a separate vial, PEO (106.4 mg, 0.0209 mmol) was dissolved in DMF (2 mL) and then added to the vial containing the backbone polymer along with additional DMF (3 mL). CuCl (4.2 mg, 0.0424 mmol) was added, and the vial was sealed with a rubber septum. The headspace of the reaction mixture was purged with nitrogen gas for 15 min, and then *N*,*N*,*N*′,*N*″,*N*″-pentamethyldiethylenetriamine (PMDETA) (10.0 μL, 0.0480 mmol) was injected using a microsyringe. After 24 h, benzyl propargyl ether (25 μL, 0.173 mmol) was injected using a microsyringe to attempt to cap unreacted azide groups. The reaction mixture was stirred for 3 h before being passed through a basic alumina (top)/silica gel (bottom) column to remove the copper catalyst. The excess side chains were removed by several rounds of centrifugal filtration using 50 kDa MWCO centrifugal filter tubes and a mixture of methanol and water (50/50, v/v) at 5000 rpm (Eppendorf 5804 Centrifuge). The methanol was removed by rotary evaporation, and the mBBs were freeze-dried using a LABCONCO 76705 Series Freeze Dryer. An additional PEO bottlebrush polymer with a grafting density of 19.4% (PEO mBB-19-2) was synthesized for

WAXD and SAXS study using an azide-functionalized backbone polymer (PHEMA-N_3-550, see Figure S1 in the Supporting Information) with a degree of polymerization of 550 and a degree of azide functionalization of 96.7% via the same procedure as for PEO mBB-47 except the use of THF instead of DMF as the solvent. The backbone polymer PHEMA-N_3-550 has an $M_{n,SEC}$ of 112.2 kDa and a Đ of 1.15 measured relative to polystyrene standards using a PL GPC-50 Plus system with GRAM columns and *N*,*N*-dimethylformamide (DMF) containing 50 mM LiBr as the eluent. From the SEC analysis of the final reaction mixture, the grafting density of PEO mBB-19-2 was calculated to be 19.4%. The $M_{n,SEC}$ and Đ of PEO mBB-19-2 were found to be 6.1×10^5 Da and 1.18, respectively, using polystyrene calibration. The unreacted PEO side chains were completely removed as shown by SEC analysis. Figure S2 shows the SEC curves for all the PEO MBBs before and after purification. The ^{1}H NMR spectra of the purified PEO MBBs in $CDCl_3$ are included in Figure S3.

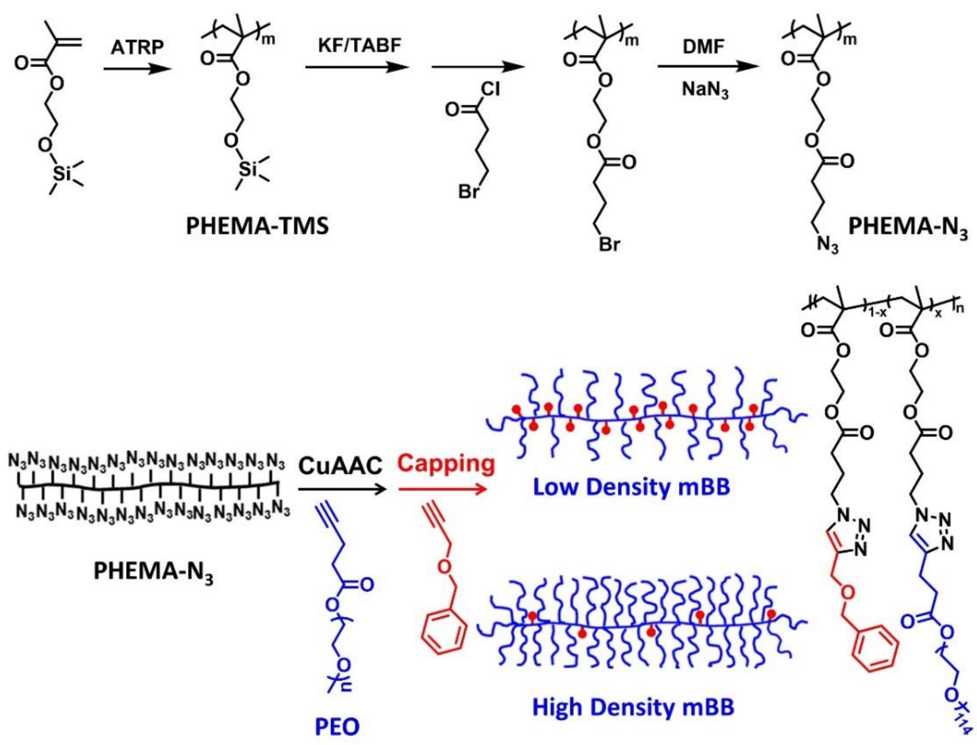

Figure S1. Synthetic scheme for PEO mBBs. The grafted side chains are anticipated to be randomly distributed along the backbone.

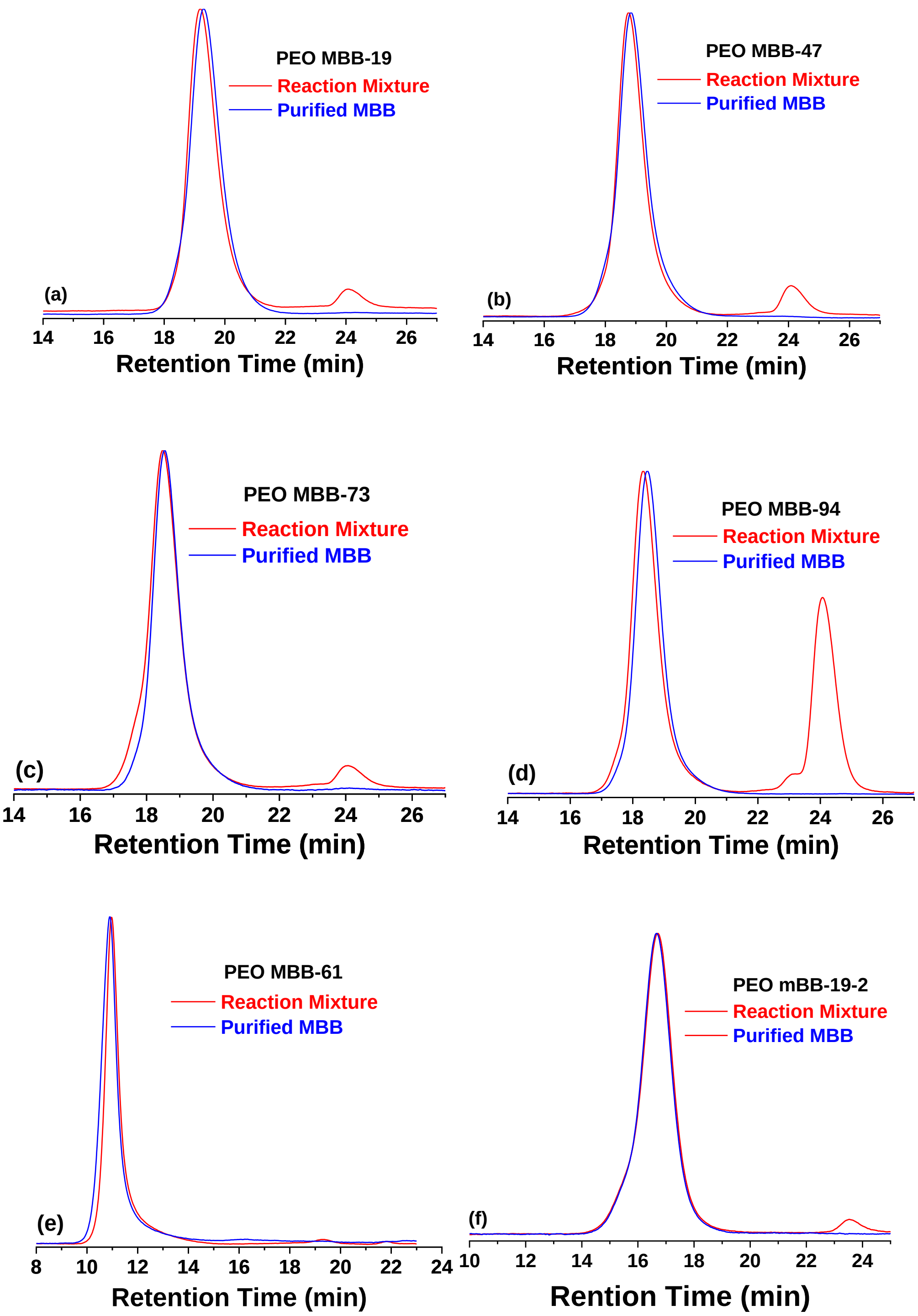

Figure S2. SEC analysis of PEO MBBs before and after purification for (a) PEO mBB-19, (b) PEO mBB-47, (c) PEO mBB-73, (d) PEO mBB-94, (e) PEO MBB-61, and (f) PEO mBB-19-2. SEC was performed with a GPC system equipped with Mixed-B columns (for PEO mBB-19, -47, -73, -94, a-d) or GRAL columns (for PEO mBB-61, e) or GRAM columns (for PEO mBB-19-2, f) and DMF with 50 mM LiBr as eluent.

(a) PEO MBB-47

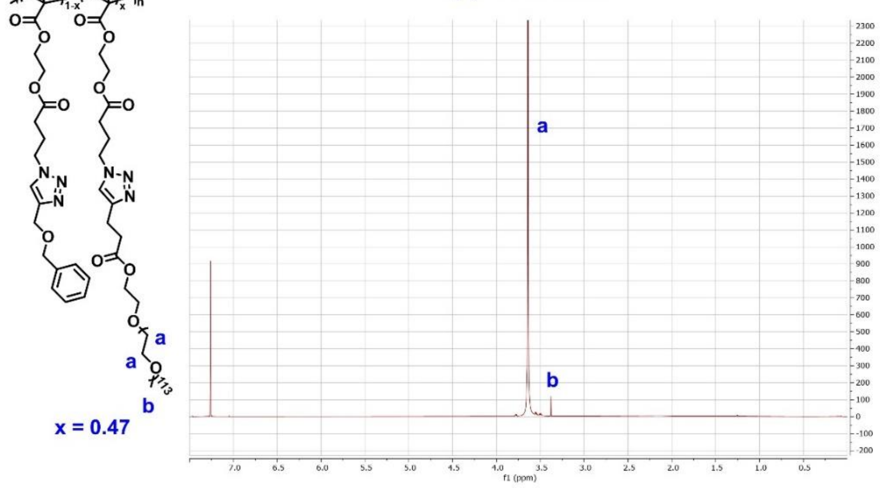

(b) PEO MBB-61

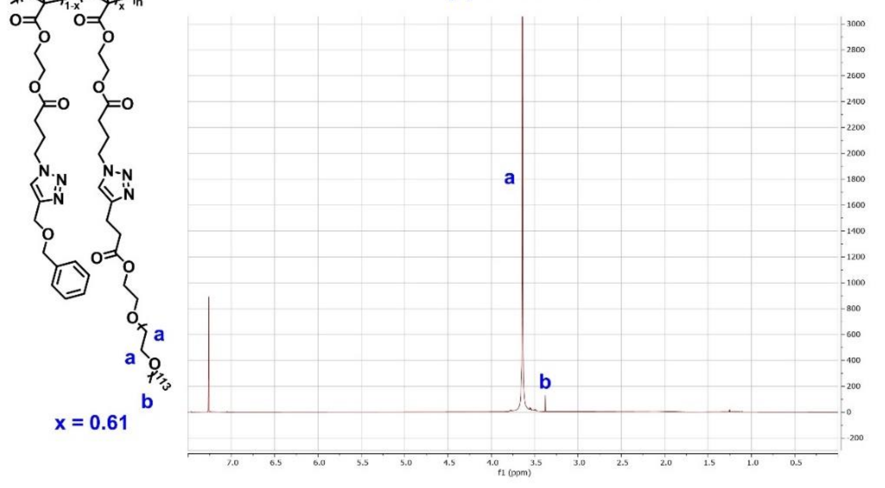

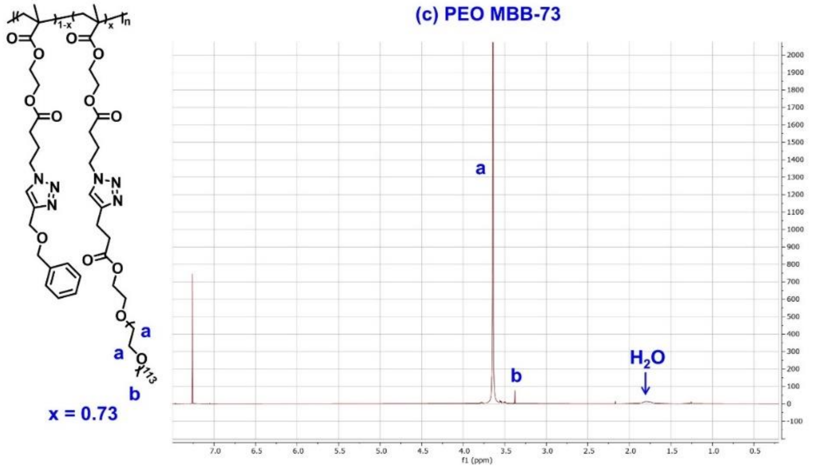

(c) PEO MBB-73
1-x
x
n
a
a
b
x = 0.73
a
b
H_2O
f1 (ppm)

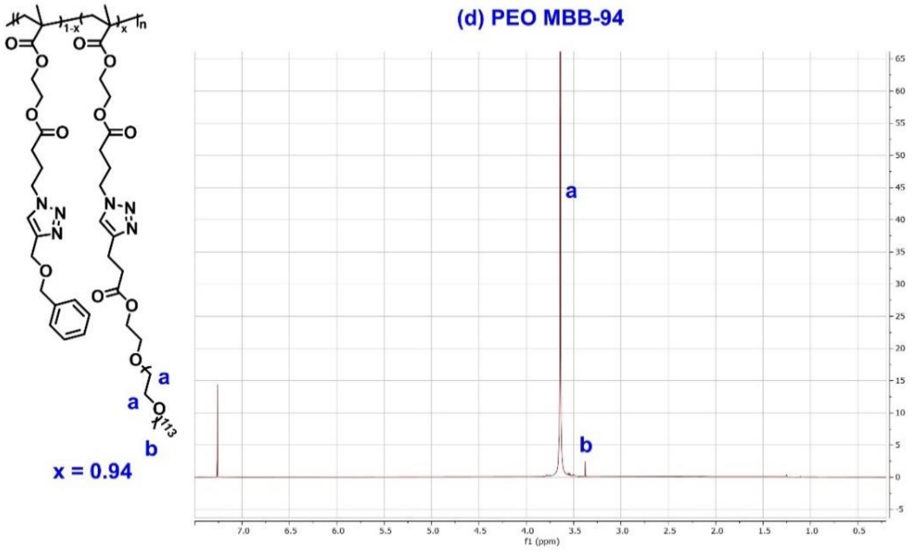

(d) PEO MBB-94
1-x
x
n
a
a
b
x = 0.94
a
b
f1 (ppm)

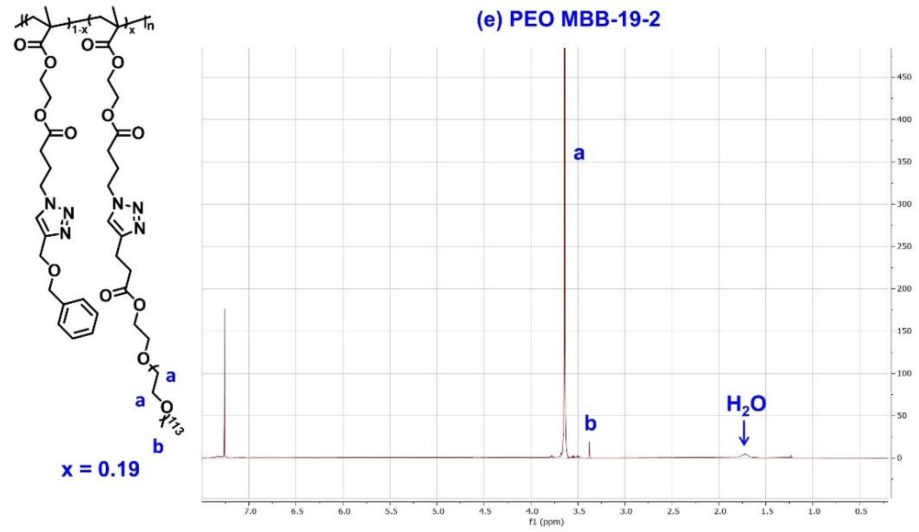

Figure S3. ^{1}H NMR spectra of PEO MBBs in $CDCl_3$: (a) PEO MBB-47, (b) PEO mBB-61, (c) PEO mBB-73, (d) PEO mBB-94, and (e) PEO mBB-19-2.

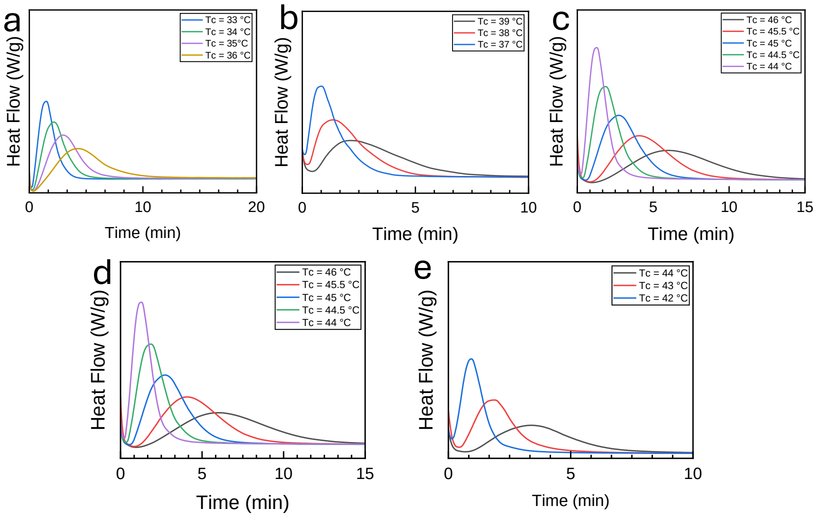

Figure S4. Isothermal crystallization exotherms for mBB samples at selected temperatures. (a) mBB-19, (b) mBB-47, (c) mBB-61, (d) mBB-73, and (e) mBB-94.

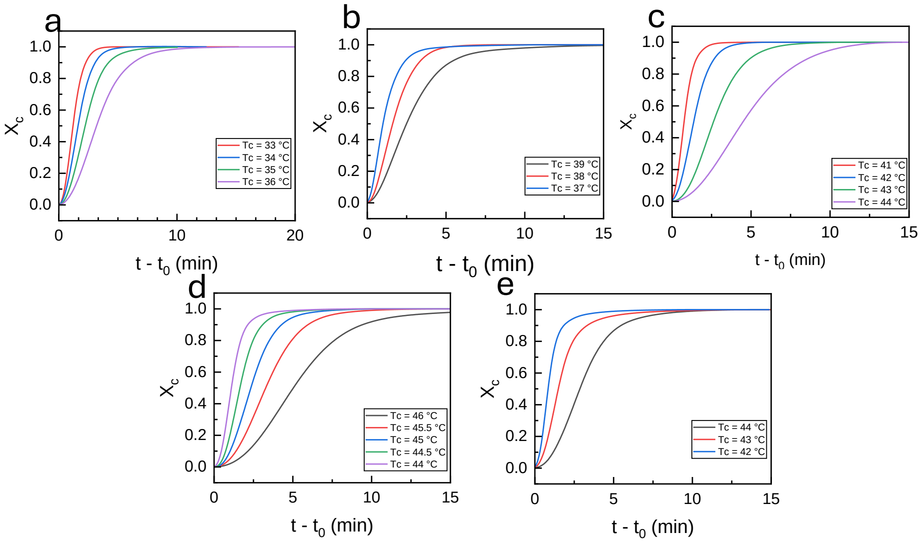

Figure S5. X_c evolution with time at different crystallization temperatures (T_c) for isothermal crystallization of mBB samples. (a) mBB-19, (b) mBB-47, (c) mBB-61, (d) mBB-73, and (e) mBB-94.

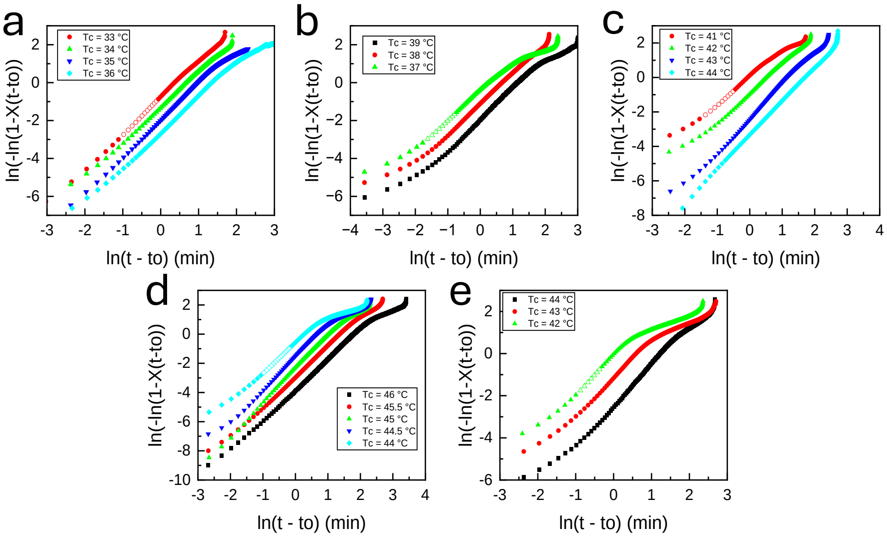

Figure S6. Avrami plots for mBB samples at selected temperatures. (a) mBB-19, (b) mBB-47, (c) mBB-61, (d) mBB-73, and (e) mBB-94. The open signals indicate representatively the data points used to obtain the Avrami exponent and the kinetic parameter K.

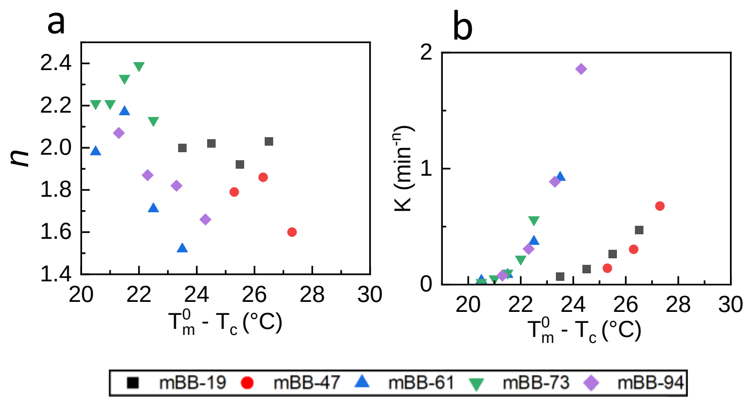

Figure S7. Plots of (a) Avrami exponent, *n*, and (b) the kinetic parameter, *K*, against undercooling (T_m^0 - T_c) of mBB-*x*.

Table S1. Summary of T_m^o for mBB samples

Sample	T_m^o (°C)
mBB-19	59.5
mBB-47	64.3
mBB-61	64.5
mBB-73	66.5
mBB-94	65.3

Table S2. Summary of T_c *and* T_m *values* for isothermal crystallization of mBBs

MBB 0.19		MBB 0.47		MBB 0.61		MBB 0.73		MBB 0.94	
T_c	T_m	T_c	T_m	T_c	T_m	T_c	T_m	T_c	T_m
°C	°C	°C	°C	°C	°C	°C	°C	°C	°C
39	54.29	44	57.15	45	57.83	46	58.14	46	58.4
38	53.91	43	56.74	44	57.49	45.5	57.96	45	58.01
37	53.75	42	56.41	43	57.07	45	57.77	44	57.67
36	53.49	41	56.04	42	56.81	44.5	57.55	43	57.26
35	53.22	40	55.64	41	56.48	44	57.34	42	56.87
		39	55.4			43.5	57.13	41	56.65

Table S3. Tabulated 10% and 50% crystallization times and Avrami parameters for mBB samples.

Sample	T_c (°C)	T_m^o - T_c (°C)	$t_{0.1}$ (min)	$t_{0.5}$ (min)	n	K (min ^-n)
mBB-19	36	23.49	1.22	3.2	2.00	0.07
	35	24.49	0.89	2.26	2.02	0.14
	34	25.49	0.61	1.64	1.92	0.26
	33	26.49	0.47	0.85	2.03	0.47
mBB-47	39	25.3	0.85	2.49	1.79	0.14
	38	26.3	0.6	1.62	1.86	0.30
	37	27.3	0.34	0.99	1.60	0.68
mBB-61	44	20.35	1.75	4.53	1.98	0.04
	43	21.35	1.11	2.65	2.17	0.09
	42	22.35	0.52	1.37	1.71	0.37
	41	23.35	0.24	0.77	1.52	0.92
mBB-73	46	20.47	2.13	5.05	2.21	0.02
	45.5	20.97	1.4	3.29	2.21	0.05
	45	21.47	1.03	2.33	2.33	0.10
	44.5	21.97	0.75	1.64	2.39	0.22
	44	22.47	0.45	1.1	2.13	0.56
mBB-94	44	21.32	1.2	2.87	2.07	0.08
	43	22.32	0.6	1.48	1.87	0.31
	42	23.32	0.32	0.83	1.82	0.89
	41	24.32	0.19	0.5	1.66	1.86

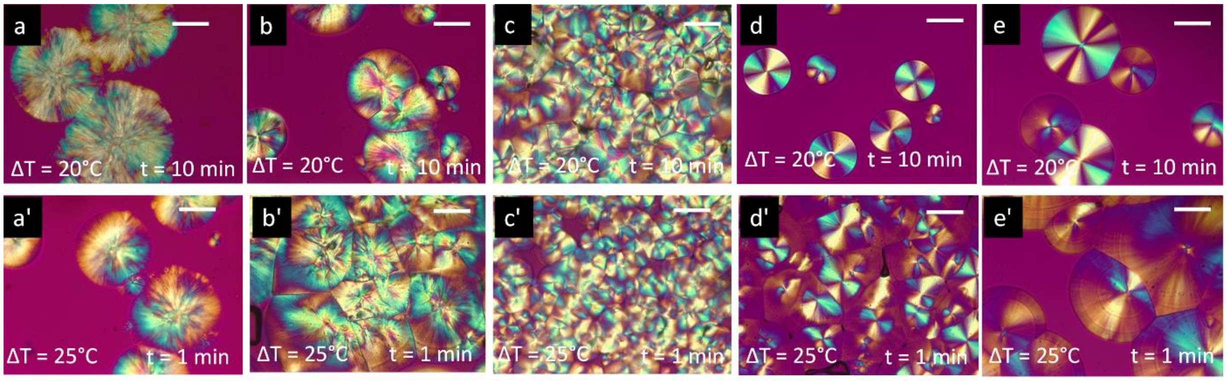

Figure S8. PLM images of spherulites crystallized at constant undercoolings. The top row shows PEO mBBs with grafting densities of (a) 0.19, (b) 0.47, (c) 0.61, (d) 0.73, and (e) 0.94 crystallized at ΔT = 20 °C for 10 min. The bottom row shows PLM images taken at ΔT = 25 °C after 1 min crystallization for PEO mBBs with the respective grafting densities. The scale bar is 40 μm.

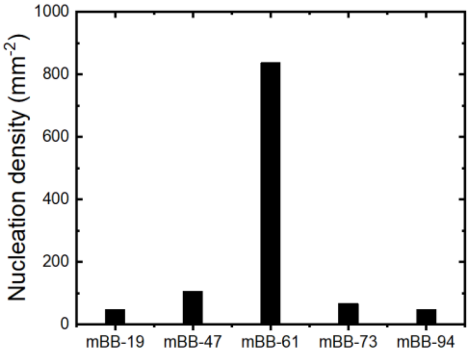

Figure S9. Nucleation density for mBBs crystallized at ΔT = 20 °C for 10 min.

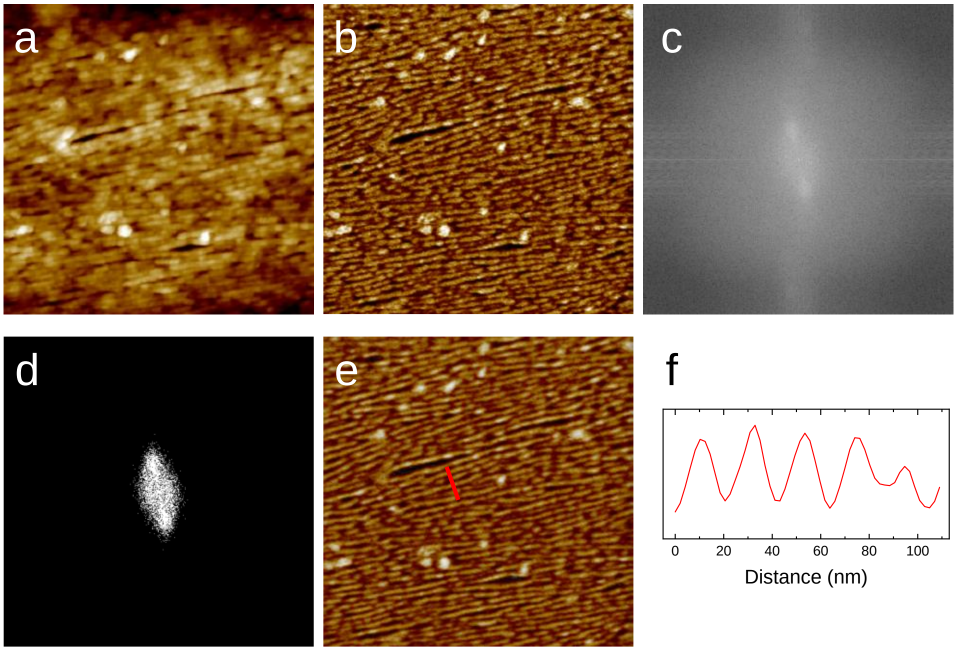

Figure S10. Fast-Fourier Transform (FFT) analysis of an acquired mBB-94 spherulite image. (a) AFM height topography image of an mBB-94 spherulite with scan dimensions of 1µm × 1µm. b) LogDMT modulus data acquired from (a) and the corresponding FFT image (c). Modulus data was chosen due to enhanced contrast between the crystalline regions. (d) Mask applied to isolate select pixels from the image in (c). (e) Reverse FFT transform containing high frequency data points converted back to a RGB image. The red line denotes the surface profile. (f) Surface profile of the smoothed image measuring pixel intensity vs surface distance.

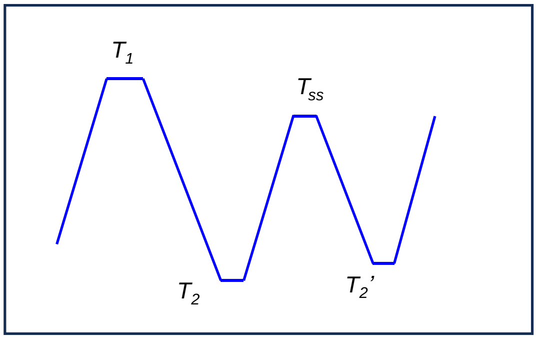

Figure S11. Schematic representation of the temperature profile used for self-seeding experiments. T_1 and T_2, 100 °C and 0 °C, respectively, were chosen as the upper and lower limits of the temperature. T_{ss} is the self-seeding temperature, and T_2' is 20 °C.

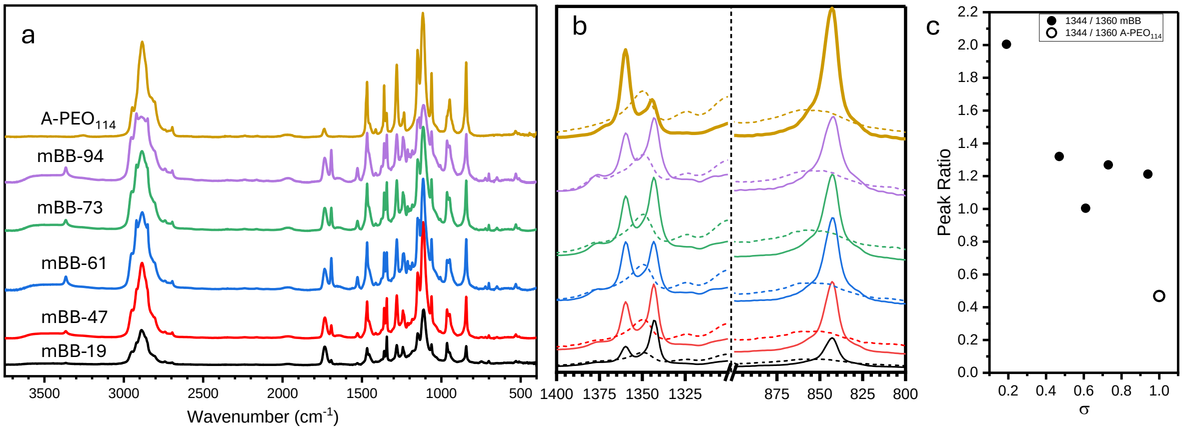

Figure S12. FTIR of mBBs. (a) FTIR spectra from 400-4000 cm^{-1} obtained at 30 °C. (b) FTIR spectra at 30 °C (solid lines) and 70 °C (dash lines) highlight the trans and helical conformation of the chain (see text). (c) Intensity ratios of the 1344/1360 cm^{-1} bands.

The conformational behavior of PEO and other polyethers has been probed by FTIR spectroscopy in blends[4-6] crystalline state[7-10] and melt state.[5] The presence and relative intensity of certain skeletal vibration bands can indicate the relative population of different conformations of PEO. The bands at 1360 (CH_2 wagging) indicate the presence of helical conformers and 1344 indicate the presence of planar zigzag / trans planar conformers.[4-6] It was found that, after crystallization, grafted PEO side chains had an increased planar zigzag to helical conformation ratio compared to linear A-PEO_{114}, as indicated by the decreases A_{1344}/A_{1360} ratio (**Figure S11c**), on the same order of magnitude as that reported by Marentette and Brown[4] upon blending with PVC. There is no evidence of the triclinic planar zigzag crystal modification of PEO in WAXD results, which indicates that this effect is localized to the conformation of individual chains. The increased trans conformation in mBB crystals is attributed to the stretching of the side chains away from the backbone upon crystallization.[5] There is no strong indication that these conformation differences persist in the melt. For example, typical CH_2 rocking for *trans* conformation can be found at 862 cm^{-1}.[5] Our results show similar intensity of the 862 cm^{-1} band for all samples (Figure S11b, right panel), suggesting that the difference of *trans*-conformation population between mBBs and A-PEO_{114} is not discernable from FTIR.

Table S4. Tabulated end of melting endotherm and T_{clear} values for mBBs with different grafting densities. The strength of the memory effect can be defined as $T_{\text{clear}} - T_{\text{end of endotherm}}$.

Sample	*End of endotherm* (°C)	T_{clear} (°C)	*DIIa width* (°C)
mBB-19	57	70	13
mBB-47	59	75	16
mBB-61	61	85	24
mBB-73	61	85	24
mBB-94	61	90	29

References

1. Kent, E. W.; Lewoczko, E. M.; Zhao, B., Effect of buffer anions on pearl-necklace morphology of tertiary amine-containing binary heterografted linear molecular bottlebrushes in acidic aqueous buffers. *Langmuir* **2020,** *36*, 13320-13330.
2. Henn, D. M.; Fu, W.; Mei, S.; Li, C. Y.; Zhao, B., Temperature-induced shape changing of thermosensitive binary heterografted linear molecular brushes between extended wormlike and stable globular conformations. *Macromolecules* **2017,** *50*, 1645-1656.
3. Kelly, M. T.; Kent, E. W.; Zhao, B., Stepwise conformational transitions of stimuli-responsive linear ternary heterografted bottlebrush polymers in aqueous solution. *Macromolecules* **2022,** *55*, 1629-1641.
4. Marentette, J. M.; Brown, G. R., The crystallization of poly(ethylene oxide) in blends with neat and plasticized poly(vinyl chloride). *Polymer* **1998,** *39*, 1415-1427.
5. Li, X.; Hsu, S. L., An analysis of the crystallization behavior of poly(ethylene oxide)/poly(methyl methacrylate) blends by spectroscopic and calorimetric techniques. *J. Polym. Sci. Polym. Phys.* **1984,** *22*, 1331-1342.
6. Rao, G. R.; Castiglioni, C.; Gussoni, M.; Zerbi, G.; Martuscelli, E., Probing the structure of polymer blends by vibrational spectroscopy: The case of poly(ethylene oxide) and poly(methyl methacrylate) blends. *Polymer* **1985,** *26*, 811-820.
7. Yoshihara, T.; Tadokoro, H.; Murahashi, S., Normal vibrations of the polymer molecules of helical conformation. Iv. Polyethylene oxide and polyethylene-d4 oxide. *J. Chem. Phys.* **1964,** *41*, 2902-2911.
8. Makino, D.; Kobayashi, M.; Tadokoro, H., Structural studies of polyethers [· (ch2)m · o ·]n · xi. Skeletal vibrations of planar zigzag polyethers. *Spectrochim. Acta, Part A* **1975,** *31*, 1481-1495.
9. Geng, Y.; Wang, G.; Cong, Y.; Bai, L.; Li, L.; Yang, C., Surface adsorption-induced conformational ordering and crystallization of polyethylene oxide. *J. Polym. Sci. Polym. Phys.* **2010,** *48*, 106-112.
10. Miyazawa, T.; Fukushima, K.; Ideguchi, Y., Molecular vibrations and structure of high polymers. Iii. Polarized infrared spectra, normal vibrations, and helical conformation of polyethylene glycol. *J. Chem. Phys.* **1962,** *37*, 2764-2776.
